# Endoscopic Spine Surgery vs. Conventional Approaches for Lumbar Spondylolisthesis: Systematic Review and Meta-Analysis

**DOI:** 10.3390/jcm15124751

**Published:** 2026-06-18

**Authors:** Miguel de Pedro Abascal, Teresa Bas, Paloma Bas, Ghassan Elgeadi Saleh, Alberto Caballero García, Joint Halley Guimbard Perez, Amparo Ortega Yago, Miguel Ángel Castillo Soriano

**Affiliations:** 1 Instituto Cirugía Avanzada de Columna, 28002 Madrid, Spain; migueldepedroa@gmail.com (M.d.P.A.); elgeadi@clinicaelgeadi.com (G.E.S.); albertocaballerogarcia@gmail.com (A.C.G.); ortegayago94@gmail.com (A.O.Y.); 2 Department of Orthopedic Surgery and Traumatology, University and Polytechnic La Fe Hospital, 46026 Valencia, Spain; pabasher@mail.ucv.es; 3 Servicio de Cirugía Ortopédica y Traumatología, Hospital Laboral Solimat, 45004 Toledo, Spain; jguimbardmdao@gmail.com; 4 Department of Orthopedic Surgery and Traumatology, Umivale Activa Valencia, 46008 Valencia, Spain; drmiguelangelcastillo@gmail.com

**Keywords:** endoscopic spine surgery, lumbar spondylolisthesis, minimally invasive surgery, transforaminal lumbar interbody fusion, systematic review and meta-analysis

## Abstract

**Background/Objectives**: To determine whether ESS provides superior clinical, radiologic, or perioperative outcomes compared with non-ESS surgical strategies in lumbar spondylolisthesis. **Methods**: We conducted a PRISMA-guided systematic review and meta-analysis comparing ESS with non-ESS strategies specifically for lumbar spondylolisthesis. PubMed, Web of Science, Scopus, and CENTRAL were searched from inception to December 2025, plus reference-list screening. Primary outcomes were mean change in VAS back pain, VAS leg pain, and Oswestry Disability Index (ODI); secondary outcomes included radiologic measures (disc height, lumbar lordosis angle, fusion rate) and perioperative outcomes (blood loss, operative time, length of stay, complications). **Results**: Eighteen studies (16 retrospective cohorts, 1 RCT, 1 case–control) involving 1200 patients with lumbar spondylolisthesis (2019–2025) were included. ESS showed no significant differences versus non-ESS in mean change in VAS back pain (13 studies; MD −0.07), VAS leg pain (14 studies; MD 0.08), or ODI (12 studies; MD 0.51). No statistically significant differences were detected in radiological outcomes (disc height, lumbar lordosis angle, and fusion rate). ESS was associated with reduced blood loss (MD −132.98) and shorter hospital stay (MD −2.86 days), with no difference in operative time (MD 3.96) or postoperative complications (RR 0.86). Subgroup analyses compared endoscopic fusion with MIS fusion, open fusion, and non-endoscopic decompression. Endoscopic versus MIS fusion showed lower blood loss (MD: −50.9 mL) and shorter hospital stay (MD: −1.4 days) but longer operative time (MD: +17.2 min), with no differences in clinical outcomes. Comparisons involving decompression and open fusion were limited by the small number of studies and should be considered exploratory. **Conclusions**: For lumbar spondylolisthesis, no statistically significant differences were detected between ESS and non-endoscopic approaches in pain, disability, radiologic outcomes, or complication rates, with potential perioperative advantages in blood loss and length of stay. However, these findings should be interpreted cautiously because the available evidence is predominantly retrospective, procedurally heterogeneous, and affected by substantial variation in follow-up duration.

## 1. Introduction

Low back pain is a leading contributor to disability worldwide and is a major driver of healthcare utilization, work loss, and reduced quality of life [1]. When symptoms arise from common lumbar degenerative conditions, such as spondylolisthesis, surgical decompression with or without fusion is frequently considered after failure of optimized conservative therapy [2]. Over the past decade, the surgical landscape has continued to shift toward less invasive approaches that aim to achieve comparable neural decompression and stabilization while minimizing collateral tissue injury [3].

Endoscopic spine surgery (ESS) is not a single uniform intervention but a spectrum of techniques that includes full-endoscopic and biportal endoscopic approaches, as well as decompression-based and interbody fusion procedures. These techniques differ in access corridor, instrumentation, technical complexity, and learning curve, which may influence operative efficiency, perioperative outcomes, and generalizability across studies [4]. This evolution has been facilitated by improvements in endoscopic optics, instrumentation, and access routes that allow targeted work through small portals with continuous irrigation and magnified visualization. Proposed advantages include reduced paraspinal muscle disruption and postoperative inflammatory response, which may translate into lower perioperative blood loss, shorter hospital stay, and faster early recovery compared with conventional non-endoscopic strategies [5]. At the same time, ESS is frequently described as technically demanding, with a steep learning curve and meaningful variability in surgeon experience, case selection, and operative workflow, factors that may affect operative time, complication profiles, and the generalizability of results across centers [6].

Despite increasing adoption, the comparative effectiveness of ESS versus non-ESS lumbar surgery remains debated. Individual studies and prior syntheses have suggested that while endoscopic and conventional approaches can both be safe and effective, differences in patient-reported outcomes, radiographic restoration, and fusion success may be small, inconsistent, or dependent on comparator type and follow-up duration [7]. Furthermore, the literature spans heterogeneous procedures, access techniques, and comparator strategies, making it challenging for clinicians to apply findings to real-world decision-making.

Accordingly, we conducted a systematic review and meta-analysis to synthesize contemporary comparative clinical evidence on ESS-based lumbar spondylolisthesis surgery versus non-ESS approaches. We focused on clinically meaningful patient-reported outcomes, alongside radiological outcomes and operative/perioperative metrics, with planned subgroup analyses by comparator type to clarify whether any effects differ when ESS is benchmarked against MIS versus open surgery.

## 2. Materials and Methods

### 2.1. Protocol Registration

Our systematic review protocol was registered in OSF (registration ID:10.17605/OSF.IO/SZ2HE). This systematic review and meta-analysis were conducted in accordance with the Preferred Reporting Items for Systematic Reviews and Meta-Analyses (PRISMA) statement and methodological guidance from the Cochrane Handbook for Systematic Reviews of Interventions (Supplementary Table S1) [8,9].

### 2.2. Data Sources and Search Strategy

We performed a comprehensive, filter-free electronic search of PubMed (MEDLINE), Web of Science, Scopus, and the Cochrane Central Register of Controlled Trials (CENTRAL) from inception to December 2025. The search strategy combined controlled vocabulary and free-text keywords related to ESS and conventional comparators, alongside terms capturing lumbar spondylolisthesis surgery. We also hand-searched the reference lists of all included studies and relevant reviews to identify additional eligible articles. The full database-specific search strategies and search yield are provided in Supplementary Table S2.

### 2.3. Eligibility Criteria

We included comparative clinical studies whether they are randomized controlled trials, cohort studies, or case–control studies enrolling human participants undergoing lumbar spondylolisthesis surgery in which an ESS-based approach was compared with non-ESS strategies. The comparator group included non-endoscopic surgical approaches such as minimally invasive surgery (MIS), open spine surgery (OSS), microscope-assisted procedures, and, in some studies, decompression-based procedures without fusion. We acknowledge that these comparators are clinically heterogeneous and may differ in surgical indication, extent of stabilization, and biomechanical objectives. Therefore, pooled estimates comparing ESS with non-ESS approaches should be interpreted as comparisons across a broad non-endoscopic surgical spectrum rather than a single uniform comparator. Studies were eligible if they reported at least one outcome of interest with extractable data for quantitative synthesis.

The primary outcomes were mean change in: (i) VAS back pain, (ii) VAS leg pain, and (iii) Oswestry Disability Index (ODI). Secondary outcomes included radiological measures (e.g., disc height [DH], lumbar lordosis angle [LLA], and interbody fusion rate) and operative/perioperative outcomes (blood loss, operative time, length of hospital stay, and postoperative complications).

We excluded non-comparative designs (case series/case reports without a comparator), non-clinical studies (cadaveric, biomechanical, in vitro), narrative reviews, systematic reviews, editorials, letters, conference abstracts, clinical guidelines, and book chapters. When overlapping or duplicate cohorts were suspected, we retained the most complete report (largest sample size, most relevant outcomes, or longest follow-up). We excluded studies that did not provide sufficient data for effect-size estimation and those published in languages other than English.

### 2.4. Study Selection and Data Extraction

All records retrieved from the databases were imported into Rayyan, v 1.7.2 (https://www.rayyan.ai/; accessed date: 1 March 2026) and duplicates were removed prior to screening. Two reviewers independently screened titles and abstracts to identify potentially relevant studies, followed by full-text assessment against the predefined eligibility criteria. Disagreements were resolved through discussion, with arbitration by a third reviewer when consensus could not be reached.

Data extraction was performed independently by paired reviewers using a piloted standardized Excel sheet. For each eligible study, we extracted: study design, country, publication year, sample size, population characteristics, surgical technique details, comparator definition, and follow-up duration. When available, baseline variables were extracted separately per group, including age, sex, and other reported clinical and radiographic characteristics.

For outcomes, we extracted continuous measures as mean change and standard deviation (SD) for VAS back pain, VAS leg pain, and ODI. Radiological outcomes included change in DH and LLA and the interbody fusion rate. Operative outcomes included estimated blood loss, operative time, length of stay, and postoperative complications.

When outcomes were reported at multiple postoperative time points, we extracted data at the longest available follow-up within each study. If dispersion measures were not directly reported, we derived SDs from available statistics using standard formulas [9]. Any discrepancies in extracted data were resolved by consensus, with adjudication by a senior reviewer when needed.

### 2.5. Risk of Bias and Certainty of Evidence

Risk of bias was assessed independently by two reviewers. For the randomized controlled trial, we used the Cochrane Risk of Bias 2 (RoB 2) tool [10], evaluating the five standard domains: (i) randomization process, (ii) deviations from intended interventions, (iii) missing outcome data, (iv) measurement of outcomes, and (v) selection of the reported results. Each outcome-specific judgement was recorded with supporting justification, and disagreements were resolved by discussion and adjudication. For non-randomized studies, we used the Newcastle–Ottawa Scale (NOS) to assess selection, comparability, and outcome/exposure domains. Studies were categorized as good, fair, or poor quality according to prespecified NOS thresholds [11].

We applied the Grading of Recommendations, Assessment, Development, and Evaluation (GRADE) approach to assess the certainty of evidence for each primary and secondary outcome. GRADE assessments were conducted for the main comparison (ESS vs. non-ESS). The certainty of evidence was rated as High, Moderate, Low, or Very Low based on the assessment of risk of bias, inconsistency, indirectness, imprecision, and publication bias. Observational studies began at low certainty and could be rated down for limitations or rated up if large effect sizes, dose–response gradients, or plausible confounding were present. The certainty assessment was incorporated into a Summary of Findings table presented in the Supplementary Materials.

### 2.6. Statistical Analysis

Statistical analyses were performed using R 4.3.3. software with meta and ggplot packages [12]. Meta-analyses were conducted using random-effects model (DerSimonian-Laird method) for all outcomes, given the inherent clinical heterogeneity expected in surgical procedure comparisons involving diverse patient populations, varying surgical techniques, and differing institutional protocols. This approach acknowledges both within-study and between-study variability, providing more conservative estimates and accounting for uncertainty in the true effect size across studies. Continuous outcomes were pooled as mean differences (MD) with 95% confidence intervals (CI), while dichotomous outcomes were pooled as risk ratios (RR) with 95% CIs. Statistical heterogeneity was evaluated using the chi-square test and quantified using I^2^; however, the decision to use random effects was based on clinical diversity rather than solely on statistical heterogeneity thresholds. Between-study heterogeneity was quantified using the τ^2^ statistic, and the magnitude of heterogeneity was interpreted by considering both statistical significance and clinical relevance, as recommended by the Cochrane methodology.

Because the non-ESS comparator encompassed multiple operative strategies with potentially different indications and biomechanical goals, and because the included interventions comprised biomechanically and clinically distinct procedures, particularly decompression-only procedures versus interbody fusion procedures, preplanned subgroup analyses were performed stratified by both comparator type and surgical objective. Two complementary subgrouping strategies were employed.

First, consistent with the original analysis plan, studies were stratified by comparator type (ESS vs. MIS and ESS vs. OSS) to explore whether the pooled effects differed across the comparator categories.

Second, to address concerns regarding the clinical heterogeneity of pooled interventions, studies were additionally stratified by surgical objective: (1) endoscopic fusion versus non-endoscopic fusion, including comparisons of endoscopic transforaminal or posterior lumbar interbody fusion techniques [such as Unilateral Biportal Endoscopic Transforaminal Lumbar Interbody Fusion (UBE-TLIF), Unilateral Biportal Endoscopic Posterior Lumbar Interbody Fusion (UBE-PLIF), Biportal Endoscopic Transforaminal Lumbar Interbody Fusion (BE-TLIF), Endoscopic Transforaminal Lumbar Interbody Fusion (Endo-TLIF), Endoscopic Transforaminal Lumbar Interbody Fusion (E-TLIF), Percutaneous Endoscopic Lumbar Interbody Fusion (PELIF), Biportal Endoscopic Extraforaminal Lumbar Interbody Fusion (BE-ELIF), Percutaneous Transforaminal Endoscopic Surgery combined with Oblique Lateral Interbody Fusion (PTES + OLIF), and Unilateral Lumbar Interbody Fusion (ULIF]) against conventional minimally invasive or open fusion procedures; (2) endoscopic fusion versus open fusion, specifically comparing endoscopic fusion techniques against conventional open fusion approaches; and (3) endoscopic decompression versus non-endoscopic decompression, including comparisons of endoscopic decompression-only procedures [such as Unilateral Laminotomy for Bilateral Decompression (ULBD), Percutaneous Endoscopic Lumbar Discectomy (PELD), Microendoscopic Muscle-preserving Interlaminar Decompression (ME-MILD), and Percutaneous Transforaminal Endoscopic Decompression (PTED]) against non-endoscopic decompression techniques. While subgroup analyses by endoscopic approach type (uniportal vs. biportal) were considered, the available data did not permit adequately powered stratified analyses.

Subgroup analyses were classified as confirmatory when ≥3 studies were available per outcome, permitting formal statistical comparisons between subgroups. Analyses with fewer studies (*n* < 3) were classified as exploratory and presented with appropriate caveats regarding the limited evidence base. Between-subgroup heterogeneity was assessed using the Q-test for heterogeneity, with *p* < 0.10 considered statistically significant. Additionally, heterogeneity within the subgroups was quantified using the I^2^ statistic, with values > 75% indicating substantial heterogeneity requiring cautious interpretation. To assess the reliability of the pooled estimates within each subgroup, leave-one-out sensitivity analyses were performed, excluding one study at a time and recalculating the pooled effects. Publication bias within subgroups was assessed using funnel plots when sufficient studies were available (≥10 studies) and statistically using Egger’s regression test. A two-sided *p*-value < 0.05 was considered statistically significant for all pooled effects; however, *p*-values for exploratory analyses should be interpreted as hypothesis-generating rather than confirmatory.

### 2.7. Confounder Assessment

Given the predominance of non-randomized study designs in our cohort, we conducted a detailed assessment of key potential confounders using the available baseline data. The variables assessed included slip grade (Meyerding classification), spondylolisthesis type (isthmic vs. degenerative), baseline symptom severity (VAS back pain, VAS leg pain, ODI), and age and sex distribution. Between-group balance was descriptively evaluated for each study. The assessment was limited by the availability of data in the primary reports; several important confounders including number of levels treated, stenosis severity, osteoporosis status, and instability assessment were not systematically reported across studies.

## 3. Results

### 3.1. Literature Search

**Figure 1:**
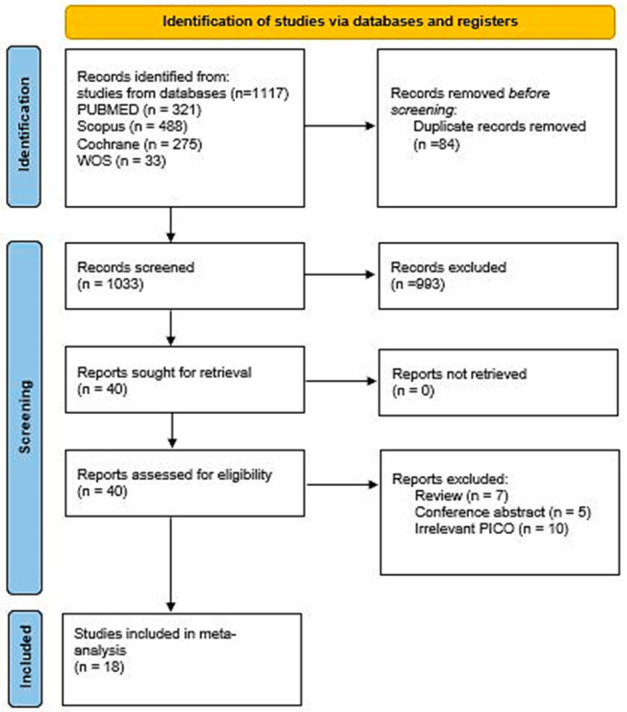
PRISMA 2020 flow diagram.

The initial literature search identified 1117 records. A total of 84 duplicate studies were removed. A total of 1033 records for title and abstract screening, followed by 40 records for full-text screening. Finally, 18 eligible articles met all inclusion criteria and were included in our study [13,14,15,16,17,18,19,20,21,22,23,24,25,26,27,28,29,30]. No additional articles were identified after manually reviewing the references from the studies included. The PRISMA flow diagram visualizes the selection process and reasons for study exclusions (Figure 1).

### 3.2. Characteristics of Included Studies

We included 18 studies (16 retrospective cohort, one RCT, and one case–control) [13,14,15,16,17,18,19,20,21,22,23,24,25,26,27,28,29,30] for quantitative analysis, with a total of 1200 patients with lumbar spondylolisthesis. All studies were published between 2019 and 2025. When reported, the mean age was 63 ± 10.4 years, with a female predominance (*n* = 576, 52.4%). Twelve studies were conducted in China, two studies in South Korea, and additional studies were conducted in Japan, Taiwan, and Singapore. The follow-up duration ranged from 12 to 81 months. Detailed summary and baseline data are reported in Table 1 and Table 2 [13,14,15,16,17,18,19,20,21,22,23,24,25,26,27,28,29,30].

A detailed confounder assessment evaluating between-group balance for key baseline variables including slip grade, spondylolisthesis type, baseline symptom severity, and radiographic parameters is presented in Supplementary Table S3.

### 3.3. Risk of Bias Assessment and Certainty of Evidence

Based on the Cochrane Risk of Bias 2 (RoB 2), one study presented some concerns (Supplementary Figure S1). The one included RCT (Lv et al., 2022) [24] was assessed using RoB 2 and rated as ‘some concerns’ primarily due to the absence of prospective trial registration (limiting verification of protocol adherence), the single-center design, and lack of participant or outcome assessor blinding—design features common in surgical trials but nonetheless introducing potential bias. Regarding NOS, 15 studies were rated as good quality and two studies as fair quality, as shown in Supplementary Table S4.

The certainty of evidence for the main comparisons, assessed using the GRADE approach, is presented in Supplementary Table S5. Overall, the certainty of evidence was rated as Very Low for all primary and secondary outcomes due to the predominance of observational study designs, substantial to high heterogeneity across studies, and wide confidence intervals including both clinically important benefit and harm for patient-reported outcomes.

### 3.4. Primary Outcomes

#### 3.4.1. Mean Change in VAS Pain Scores

**Figure 2:**
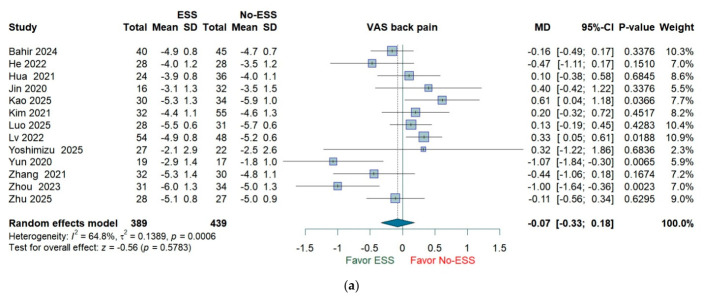

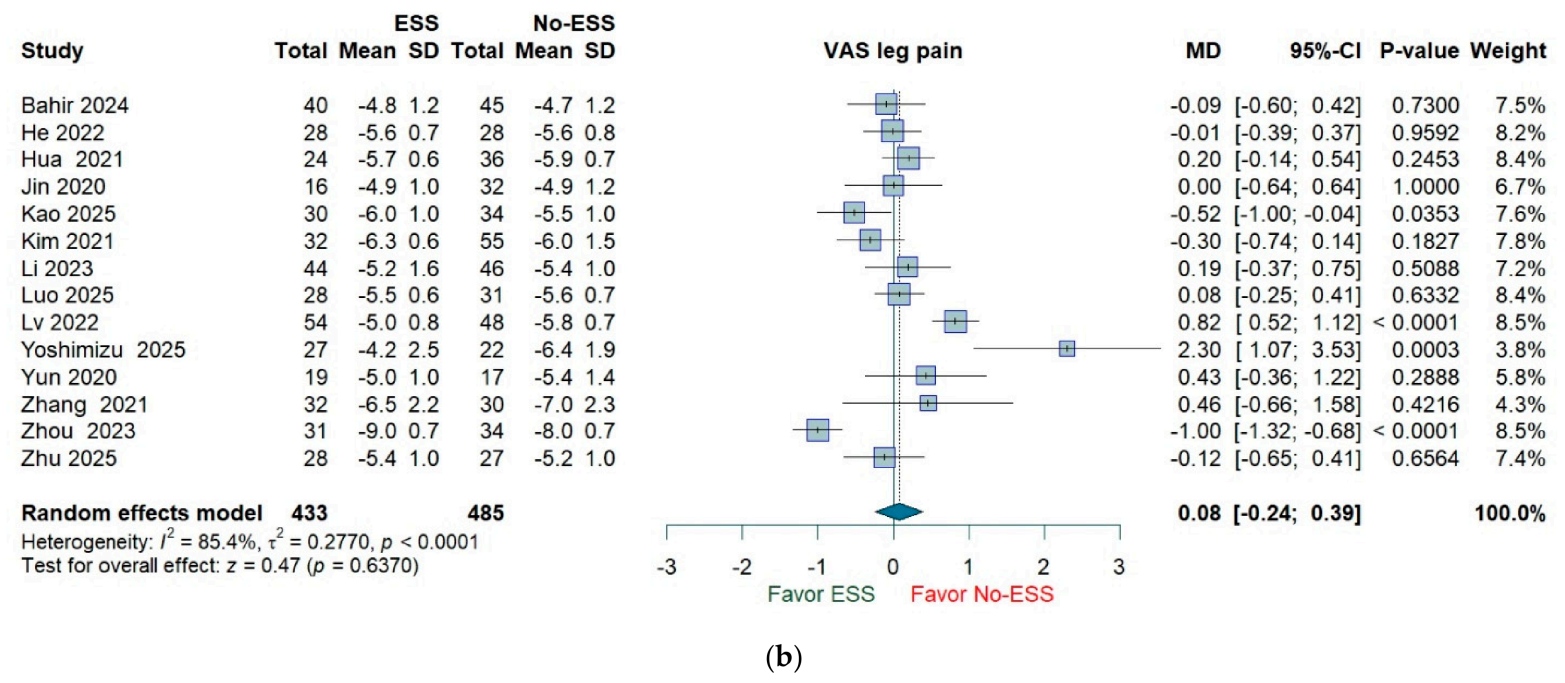
(**a**): Forest plot of VAS back pain. (**b**): Forest plot of VAS leg pain [13,14,16,17,18,19,21,22,24,26,27,28,29,30].

Thirteen studies assessed mean change in VAS back pain with 828 patients, of which the pooled estimate showed no statistically significant difference in mean change in VAS back pain between ESS and non-ESS groups (MD = −0.07, 95% CI: −0.33 to 0.18, *p* = 0.57; I^2^ = 64.8%) (Figure 2). A leave-one-out sensitivity analysis was conducted to assess the individual effect of each study by excluding one study at a time; the results remained consistent across sensitivity analyses (Supplementary Figure S2). Funnel plot was conducted, revealing some asymmetry around the pooled estimate (Supplementary Figure S3). However, Egger’s test found no significant publication bias (*p* = 0.21). Subgroup analysis based on comparator group, the pooled estimate showed no significant difference between ESS and MIS (MD = 0.01, 95% CI: −0.26 to 0.29, *p* = 0.92; I^2^ = 61.8%) or between ESS and OSS (MD = −0.40, 95% CI −1.09 to 0.29; *p* = 0.25; I^2^ = 78.5%) (Supplementary Figure S4).

**Figure 3:**
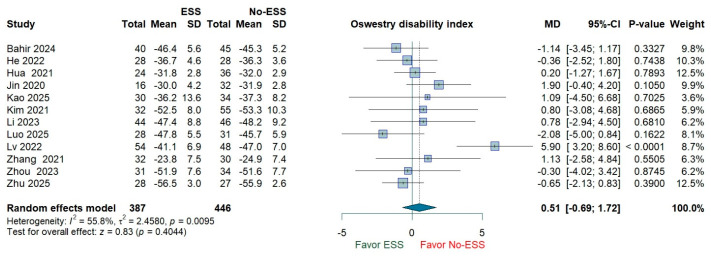
Forest plot of ODI [13,14,16,17,18,19,21,22,24,28,29,30].

14 studies assessed mean change in VAS leg pain with 918 patients, of which the pooled estimate showed no significant difference between ESS and non-ESS (MD = 0.08, 95% CI: −0.24 to 0.39, *p* = 0.63; I^2^ = 85.4%) (Figure 3). A leave-one-out sensitivity analysis was conducted to find the single effect of each study, and it was done by excluding one study at a time; the results remained consistent across sensitivity analyses (Supplementary Figure S5). Funnel plot was conducted, revealing some asymmetry around the pooled estimate (Supplementary Figure S6). However, Egger’s test found no significant publication bias (*p* = 0.49). Subgroup analysis based on comparator group, the pooled estimate showed no significant difference between ESS and MIS (MD = 0.08, 95% CI: −0.34 to 0.49, *p* = 0.92; I^2^ = 61.8%) or between ESS and OSS (MD = 0.08, 95% CI −0.16 to 0.31; *p* = 0.52; I^2^ = 78.5%) (Supplementary Figure S7).

#### 3.4.2. Mean Change in Oswestry Disability Index (ODI)

**Figure 4:**
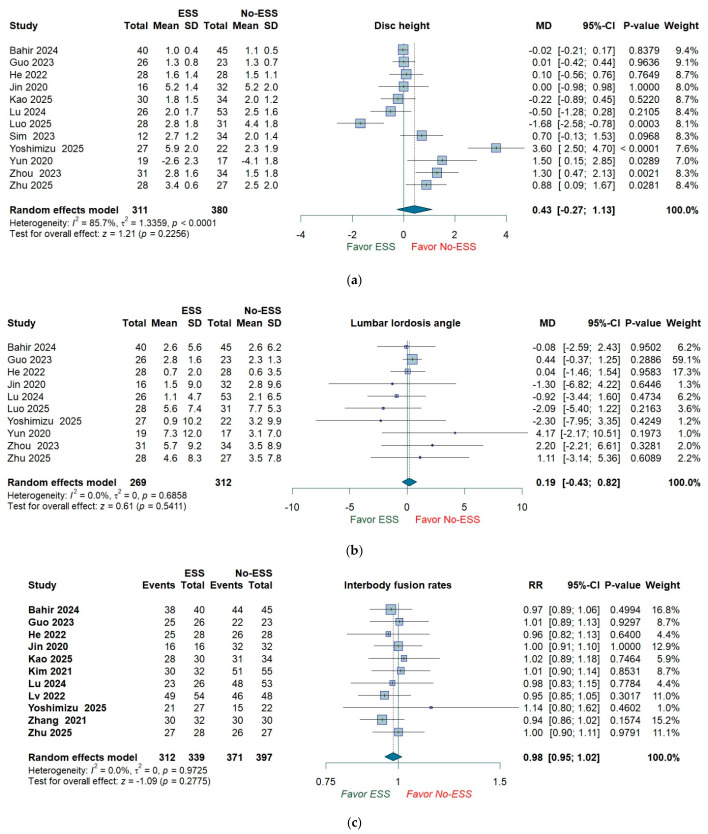
(**a**): Forest plot of disc height. (**b**): Forest plot of Lumbar lordosis angle. (**c**): Forest plot of interbody fusion rates [13,14,15,16,17,18,19,21,22,23,24,25,26,27,28,29,30].

12 studies assessed mean change in ODI with 833 patients, of which the pooled estimate showed no significant difference between ESS and non-ESS (MD = 0.51, 95% CI: −0.69 to 1.72, *p* = 0.40; I^2^ = 55.8%) (Figure 4). A leave-one-out sensitivity analysis was conducted to find the single effect of each study, and it is done by excluding one study at a time; the results remained consistent across sensitivity analyses (Supplementary Figure S8). Funnel plot was conducted with no asymmetry around the pooled estimate (Supplementary Figure S9). Moreover, Egger’s test found no significant publication bias (*p* = 0.46). Subgroup analysis based on comparator group, the pooled estimate showed no significant difference between ESS and MIS (MD = 0.89, 95% CI: −0.48 to 2.25, *p* = 0.20; I^2^ = 58.2%) or between ESS and OSS (MD = −0.97, 95% CI −2.70 to 0.77; *p* = 0.27; I^2^ = 0%) (Supplementary Figure S10).

#### 3.4.3. Sensitivity Analysis: Follow-Up Duration

To assess the potential impact of variable follow-up duration on primary outcomes, we performed sensitivity analyses stratified by follow-up duration: short-term (≤24 months, 15 studies) and long-term (>24 months, three studies). The results are listed in Table S6.

For short-term follow-up, no significant differences were detected between the ESS and non-ESS groups for VAS back pain (MD: 0.04, 95% CI: −0.14 to 0.21, *p* = 0.66, I^2^ = 27.0%), VAS leg pain (MD: 0.20, 95% CI: −0.08 to 0.48, *p* = 0.16, I^2^ = 73.0%), or ODI (MD: 0.55, 95% CI: −0.72 to 1.81, *p* = 0.40, I^2^ = 63.6%). Significant advantages for ESS were observed for hospital stay (MD: −2.86 days, 95% CI: −4.48 to −1.24, *p* = 0.0005) and blood loss (MD: −126.5 mL, 95% CI: −162.8 to −90.2, *p* < 0.0001).

Only two studies provided data on pain outcomes for long-term follow-up. The VAS back pain (MD: −0.21, 95% CI: −1.86 to 1.44, *p* = 0.80, I^2^ = 91.5%) and VAS leg pain (MD: −0.10, 95% CI: −1.02 to 0.83, *p* = 0.83, I^2^ = 75.0%) showed no significant differences, although the very limited number of studies precluded definitive conclusions. No studies with >24 months of follow-up reported operative time, blood loss, hospital stay, or ODI outcomes.

The broadly similar effect estimates for patient-reported outcomes across follow-up durations suggest that differential follow-up is unlikely to be a major source of bias in the primary outcome comparisons. However, the limited long-term data restricts conclusions about extended follow-up periods.

### 3.5. Secondary Outcomes

#### 3.5.1. Radiological Outcomes

The pooled estimate showed no significant difference between the two study groups in the change in DH (MD = 0.43, 95% CI: −0.27 to 1.13, *p* = 0.22; I^2^ = 85.7%) and LLA (MD = 0.19, 95% CI: −0.43 to 0.82, *p* = 0.54; I^2^ = 0%). The pooled analysis showed no significant difference between ESS and non-ESS regarding Interbody fusion rate (RR = 0.98, 95% CI: 0.95 to 1.02, *p* = 0.27; I^2^ = 0%).

To address potential heterogeneity arising from different imaging modalities and fusion definitions, we performed sensitivity analyses stratifying studies by imaging modality used for fusion assessment, disc height, and lumbar lordosis angle. Studies were categorized as using (i) computed tomography (CT) imaging alone, (ii) plain radiography alone, or (iii) either modality. For each modality stratum, pooled effect estimates were recalculated for the interbody fusion rates. No statistically significant differences in fusion rates were observed between the ESS and non-ESS groups, regardless of the imaging modality used. For CT-based studies, the pooled RR was 0.96 (95% CI: 0.92–1.02, *p* = 0.18, I^2^ = 0%); for X-ray-based studies, the RR was 1.02 (95% CI: 0.93–1.11, *p* = 0.73, I^2^ = 0%); and for studies using either modality, the RR was 0.99 (95% CI: 0.90–1.09, *p* = 0.84, I^2^ = 0%). The absence of heterogeneity within the modality strata (I^2^ = 0%) suggests that the imaging modality did not substantively contribute to the between-study variability in fusion outcomes. Detailed fusion definitions and classification systems employed in each study are provided in Supplementary Table S7. Three imaging modality strata were analyzed in the sensitivity analysis for disc height: CT imaging alone, plain radiography alone, and studies using either CT or X-ray. No statistically significant differences were observed between ESS and non-ESS in any stratum (CT: MD +2.21 mm, 95% CI −0.45 to 4.88, *p* = 0.104, I^2^ = 93.6%; X-ray: MD +0.21 mm, 95% CI −0.69 to 1.11, *p* = 0.651, I^2^ = 65.1%; X-ray or CT: MD −0.06 mm, 95% CI −0.38 to 0.27, *p* = 0.735, I^2^ = 0%). Regarding the sensitivity analysis of the lumbar lordosis angle, two imaging modality strata were analyzed: CT imaging alone and studies using either X-ray or CT. No statistically significant differences were observed between the groups in either stratum (CT: MD −0.12°, 95% CI −3.52 to 3.27, *p* = 0.943, I^2^ = 0%; X-ray or CT: MD +0.35°, 95% CI −0.37 to 1.06, *p* = 0.338, I^2^ = 0%).

#### 3.5.2. Operative Outcomes

The ESS showed a significant decrease in blood loss compared to non-ESS (MD = −132.98, 95% CI: −227.97 to −37.99, *p* = 0.006; I^2^ = 98.5%) (Supplementary Figure S11) and a decrease in the length of hospital stay (MD = −2.86, 95% CI: −4.51 to −1.21, *p* = 0.0007; I^2^ = 96.9%) (Supplementary Figure S12). Conversely, we found no significant differences between the ESS and non-ESS groups for operative time (MD = 3.96, 95% CI: −22.59 to 30.50, *p* = 0.77; I^2^ = 98.5%) (Supplementary Figure S13). The pooled estimate also showed no significant difference between ESS and non-ESS regarding postoperative complication (RR = 0.86, 95% CI: 0.52 to 1.44, *p* = 0.57; I^2^ = 0%) (Supplementary Figure S14). For detailed complication categorization, we performed meta-analyses for six predefined complication categories: dural tear, hematoma, infection, neurological deficit, reoperation, and revision surgery. Across all complication categories, no statistically significant differences were observed between ESS and non-ESS groups. Dural tear occurred in 7 of 327 ESS patients versus 7 of 338 non-ESS patients (RR 1.01, 95% CI 0.38–2.74, *p* = 0.978, I^2^ = 0%). Hematoma was reported in 4 of 334 ESS patients versus 3 of 403 non-ESS patients (RR 1.36, 95% CI 0.49–3.72, *p* = 0.556, I^2^ = 0%). Surgical site infection occurred in 0 of 421 ESS patients versus 8 of 519 non-ESS patients (RR 0.64, 95% CI 0.25–1.63, *p* = 0.348, I^2^ = 0%). Neurological deficit was reported in 6 of 479 ESS patients versus 9 of 581 non-ESS patients (RR 1.03, 95% CI 0.48–2.22, *p* = 0.936, I^2^ = 0%). Reoperation occurred in 7 of 356 ESS patients versus 16 of 432 non-ESS patients (RR 0.81, 95% CI 0.35–1.84, *p* = 0.609, I^2^ = 0%). Revision surgery was reported in 7 of 356 ESS patients versus 16 of 432 non-ESS patients (RR 0.81, 95% CI 0.35–1.84, *p* = 0.609, I^2^ = 0%). The absence of heterogeneity in all complication categories (I^2^ = 0%) indicates consistent findings across studies and supports the robustness of these comparative safety data.

### 3.6. Subgroup Analysis Results

#### 3.6.1. Endoscopic Fusion vs. MIS Fusion

Thirteen studies comparing endoscopic fusion techniques with MIS fusion were identified [13,14,15,16,18,22,23,24,25,26,28,29,30]. Pooled analysis demonstrated no significant differences in clinical outcomes, including VAS back pain (MD: 0.15, 95% CI: −0.04 to 0.35, *p* = 0.124, I^2^ = 35.4%) (Supplementary Table S8 and Supplementary Figure S15), VAS leg pain (MD: 0.001, 95% CI: −0.38 to 0.39, *p* = 0.997, I^2^ = 81.4%) (Supplementary Table S8 and Supplementary Figure S16), and ODI (MD: 0.77, 95% CI: −1.24 to 2.78, *p* = 0.453, I^2^ = 75.1%) (Supplementary Table S8 and Supplementary Figure S17). However, significant advantages were observed for endoscopic fusion in terms of blood loss (MD: −50.9 mL, 95% CI: −76.8 to −25.1, *p* < 0.001, I^2^ = 97.1%) and hospital stay (MD: −1.4 days, 95% CI: −2.7 to −0.1, *p* = 0.030, I^2^ = 91.1%), with a longer operative time (MD: +17.2 min, 95% CI: 0.7 to 33.6, *p* = 0.041, I^2^ = 96.6%) (Supplementary Table S8).

#### 3.6.2. Endoscopic Fusion vs. Open Fusion

Only one study compared endoscopic fusion (PE-PLIF with ULBD) against open PLIF, precluding formal meta-analysis [17]. Individual study results favored endoscopic fusion for blood loss reduction and postoperative recovery, though operative time was longer. These findings should be interpreted as exploratory due to the limited number of studies.

#### 3.6.3. Endoscopic Decompression vs. Non-Endoscopic Decompression

Two studies (Kimura 2019; Yun 2020) compared endoscopic or microendoscopic decompression techniques with conventional non-endoscopic surgical approaches [20,27]. Individual study results are presented in Supplementary Table S9. The heterogeneity in surgical techniques and comparison groups precludes definitive conclusions, and these findings are considered exploratory.

## 4. Discussion

Our meta-analysis suggests that, across contemporary comparative studies of lumbar spondylolisthesis surgery, endoscopic spine surgery (ESS) achieves no statistically significant difference in patient-reported improvement in pain and disability compared to non-ESS comparators, while showing significantly reduced blood loss and shorter hospital stay. This overall pattern is broadly consistent with the direction of effect reported in the wider lumbar endoscopy literature. When ESS is compared with minimally invasive transforaminal/posterior lumbar interbody fusion (MIS-TLIF/PLIF) or open techniques, most analyses showed no statistically significant differences in VAS and ODI improvements, with reduced tissue trauma translating into less blood loss and faster early recovery, and with operative time often being similar or longer early in the adoption phase [31,32]. Nevertheless, the interpretability of these pooled effects is constrained by the observational nature of most included studies and by important clinical heterogeneity across both the ESS and non-ESS groups.

An interesting explanation for the “equivalent symptoms, faster recovery” profile is that ESS primarily changes the surgical corridor rather than the therapeutic target. In many degenerative indications, the key determinant of medium-term pain relief and disability improvement is adequate decompression and/or stable fusion rather than incision size alone. Accordingly, several comparative studies and meta-analyses have reported that endoscopic and non-endoscopic strategies show no statistically significant differences in 6–12 months ODI/VAS trajectories when the index pathology is addressed effectively [31,33,34]. In contrast, perioperative endpoints are more sensitive to approach-related tissue disruption and perioperative care pathways, which likely explains why our pooled analyses found meaningful reductions in blood loss and hospital stay in the ESS arm despite null differences in the primary patient-reported outcomes.

Mechanistically, ESS and other minimally invasive corridors are designed to reduce paraspinal muscle stripping, denervation, and prolonged retraction pressure, factors linked to postoperative pain, muscle atrophy, and slower mobilization [35]. Biomarker studies in lumbar surgery have shown that less invasive approaches can be associated with lower perioperative signals of muscle damage as CK, myoglobin, and systemic inflammatory response, supporting the biological evidence of reduced blood loss and faster early recovery even when longer-term functional scores are similar [36]. This concept also aligns with modern comparisons among minimally invasive options: for example, meta-analyses comparing OLIF to MIS-TLIF frequently report less blood loss and shorter length of stay with no statistically significant differences in functional outcomes, underscoring that approach-related morbidity can vary independently of later symptom improvement [37]. This interpretation should be considered in light of potential case-selection effects, as ESS cohorts in observational studies may represent a more favorable surgical population.

At the same time, the very high heterogeneity we observed for several operative outcomes, including blood loss and hospital stay, is expected in this field and should temper overgeneralization. ESS is not a single uniform intervention; it includes uniportal full-endoscopic, biportal (UBE/BESS), and hybrid endoscopic-assisted approaches, with variable indications (decompression vs. interbody fusion), levels treated, instrumentation strategies, and perioperative pathways [38,39]. Pooling these techniques together may obscure important differences between ESS subtypes and limit the precision with which any single endoscopic strategy can be evaluated. Recent comparative and network analyses illustrate that these endoscopic subtypes can differ in operative time profiles, learning curves, and perioperative metrics while still producing similar patient-reported improvements overall [32]. Additionally, surgeon experience and institutional volume are likely major effect modifiers. Contemporary evidence on learning curves in endoscopic lumbar interbody fusion and unilateral biportal endoscopy highlights that operative efficiency, complication patterns, and fluoroscopy can change substantially after proficiency thresholds are reached, which could widen between-study variability when pooled across mixed experience levels [6]. Accordingly, the observed similarity between ESS and non-ESS approaches should not be interpreted as strict equivalence between ESS and any specific conventional procedure, but rather as an average comparative effect across a heterogeneous set of non-endoscopic surgical strategies.

To further explore the observed heterogeneity, we performed prespecified subgroup analyses stratified by the surgical objective. The endoscopic fusion versus MIS fusion comparison demonstrated more homogeneous results for certain outcomes (e.g., VAS back pain: I^2^ = 35.4%) than the overall pooled analysis, supporting the validity of stratification by surgical objective. Specifically, this subgroup analysis revealed that no statistically significant differences in clinical outcomes were detected between endoscopic fusion techniques and MIS fusion, while showing significantly reduced blood loss (MD: −50.9 mL, 95% CI: −76.8 to −25.1, *p* < 0.001) and shorter hospital stay (MD: −1.4 days, 95% CI: −2.7 to −0.1, *p* = 0.030), though with a longer operative time (MD: +17.2 min, 95% CI: 0.7 to 33.6, *p* = 0.041). Substantial heterogeneity persisted within this subgroup for operative time, blood loss, and hospital stay outcomes, likely reflecting variations in surgeon experience, learning curve effects, and institutional protocols rather than fundamental differences in procedural efficacy. The endoscopic decompression versus non-endoscopic decompression comparison was limited to only two studies, and the endoscopic fusion versus open fusion comparison included only one study; pooled estimates for these comparisons are presented as exploratory findings rather than confirmatory conclusions owing to the insufficient number of studies.

### 4.1. Clinical Implications

From a patient-centered standpoint, our results indicate no statistically significant differences between ESS and MIS/open comparators in back pain, leg pain, or ODI improvements, while potentially improving early recovery metrics, an advantage that may be particularly relevant for older patients and those with higher comorbidity burdens where minimizing blood loss and accelerating mobilization are priorities. When examining the primary subgroup (endoscopic fusion vs. MIS fusion specifically), these perioperative advantages were confirmed with statistically significant reductions in blood loss and hospital stay. However, operative time was significantly longer for endoscopic fusion techniques, suggesting a trade-off between surgical invasiveness and operative efficiency that may evolve as techniques mature and surgeon proficiency increases. For the endoscopic decompression and endoscopic fusion versus open fusion comparisons, limited study numbers preclude definitive clinical recommendations; these findings should be considered exploratory pending additional comparative evidence. This is also consistent with broader evidence in endoscopic lumbar procedures as discectomy or decompression suggesting comparable effectiveness and, in some analyses, lower overall complication risk versus conventional approaches, although results vary by indication and study design [40]. From a health-system perspective, even modest reductions in length of stay can translate into meaningful resource savings and capacity gains; however, shorter hospital stay is strongly influenced by perioperative protocols. Evidence syntheses of enhanced recovery after surgery (ERAS) in spine surgery show that standardized pathways can reduce length of stay and complications, meaning that part of ESS’s apparent LOS advantage across studies may reflect co-interventions rather than the endoscope alone [41].

Important trade-offs remain. Endoscopic approaches often require specialized equipment and training, and radiation exposure is a recurring concern given the reliance on fluoroscopic guidance in many endoscopic workflows. Recent work in endoscopic lumbar procedures suggests fluoroscopy-associated doses can be relatively low in experienced hands, but cumulative exposure and variability across techniques underscore the need for rigorous radiation-minimization practices and consideration of enabling technologies where appropriate [42]. This is especially relevant as learning-curve effects may transiently increase fluoroscopy time and operative duration early in adoption.

The stratified analysis of postoperative complications by type provides important reassurance regarding the safety profile of ESS relative to non-ESS approaches. Across six predefined complication categories (dural tear, hematoma, infection, neurological deficit, reoperation, and revision surgery) no statistically significant differences were observed between groups, with consistent findings across all categories (I^2^ = 0%). These results suggest that ESS does not confer increased risk of any specific complication type compared with conventional surgical approaches, which is particularly relevant given the theoretical concern that endoscopic techniques may be associated with a steeper learning curve and potentially higher rates of intraoperative complications.

### 4.2. Limitations

Our study also has several limitations that should shape interpretation. First, the evidence base included was predominantly composed of retrospective cohort studies, which are inherently susceptible to selection bias and residual confounding. In routine clinical practice, endoscopic spine surgery (ESS) may be preferentially offered to patients with less complex pathology, lower-grade spondylolisthesis, or more favorable anatomical characteristics. This potential imbalance in baseline case complexity may bias comparative outcomes in favor of ESS and cannot be fully accounted for in pooled analyses of non-randomized studies. Second, the non-ESS comparator group was clinically heterogeneous, encompassing minimally invasive fusion, open fusion, microscope-assisted procedures, and decompression-only operations in some studies. These procedures do not represent a single therapeutic strategy and may differ substantially in invasiveness, stabilization goals, and expected radiologic or perioperative outcomes. As a result, some pooled comparisons likely reflect between-procedure heterogeneity in addition to the effect of endoscopic versus non-endoscopic access itself. Third, ESS was necessarily analyzed as a single umbrella category despite encompassing multiple distinct techniques, including uniportal and biportal approaches, microendoscopic procedures, decompression-only operations, and endoscopic fusion strategies. To address this limitation, we performed subgroup analyses stratified by surgical objective, distinguishing between endoscopic versus MIS fusion, endoscopic versus open fusion, and endoscopic versus non-endoscopic decompression. While subgroup analyses by endoscopic approach type (uniportal vs. biportal) were considered, the available data did not permit adequately powered stratified analyses due to ambiguous classification of portal configuration in several primary studies. Furthermore, our subgroup analyses stratifying by surgical objective demonstrated that this variable more directly explains between-study heterogeneity (I^2^ decreased from 64.8% to 35.4% when isolating fusion-only comparisons), suggesting that portal configuration is less likely the primary driver of heterogeneity in this literature. Future trials should explicitly report approach type and surgical objectives to enable more granular analyses of potential effect modifiers. Future trials should explicitly report the approach type to enable more granular subgroup analyses. These procedures differ not only in operative workflow, instrumentation, and learning curve demands, but also in their therapeutic objectives, as decompression-only procedures and interbody fusion procedures are not biomechanically equivalent interventions. Accordingly, the pooled ESS estimates should be interpreted as average effects across heterogeneous endoscopic-based strategies rather than as the effect of a single standardized procedure, and differences between ESS and comparators should be interpreted as statistical findings rather than as evidence of equivalence.

Fourth, heterogeneity was high for several endpoints, particularly blood loss, operative time, and length of stay, likely reflecting differences in ESS subtype, pathology mix, comparator definitions, surgeon experience, and perioperative protocols, and it means pooled point estimates should be interpreted as average effects across a heterogeneous landscape rather than precise predictions for any single setting. Fifth, while we conducted a detailed assessment of available baseline characteristics to evaluate potential confounding by indication, several clinically relevant confounders were not systematically reported in the primary studies, including number of levels treated, osteoporosis status, and radiographic instability assessment. This limits the ability to fully characterize case-mix differences between ESS and comparator groups, and residual confounding cannot be excluded given the observational nature of the included evidence. Sixth, while we applied the GRADE framework to assess certainty of evidence, the Very Low certainty ratings for all outcomes reflect the fundamental limitations of the available evidence base. The predominance of retrospective observational studies, combined with substantial heterogeneity and imprecise effect estimates, means that we have very little confidence in the effect estimates. Future well-designed randomized trials would be needed to increase certainty in these findings. Finally, while our publication bias tests were not statistically significant for the primary outcomes, funnel asymmetry in some analyses and language restrictions leave open the possibility of selective publication and reporting. For subgroup analyses with fewer than three studies per outcome, pooled estimates are presented as exploratory rather than confirmatory findings, consistent with established methodological recommendations for meta-analytic subgroup analysis. Taken together, the combined effects of selection bias, procedural heterogeneity, variable follow-up duration, and the limitations of the underlying study designs reduce the interpretability of the pooled estimates and preclude definitive conclusions regarding comparative effectiveness.

### 4.3. Future Opportunities

Future research should prioritize well-designed, adequately powered randomized trials and prospective registries that stratify by ESS subtype (for example uniportal vs. biportal), standardize outcome measurement and follow-up windows, and transparently report surgeon experience, learning-curve phase, and radiation metrics. Comparative effectiveness studies that include other minimally invasive comparators such as OLIF/LLIF or MIS-TLIF variants can help clarify where ESS offers a distinct advantage versus being one of several minimally disruptive corridors with broadly similar patient-reported outcomes [37]. In parallel, cost-effectiveness analyses are needed to weigh capital and disposables against potential savings from reduced length of stay and faster functional recovery, ideally within ERAS-standardized pathways to isolate the incremental value of the endoscopic approach [41]. Finally, mechanistic work linking quantitative measures of muscle preservation (imaging-based multifidus quality, biomarkers of muscle injury/inflammation) to patient-important recovery endpoints could clarify which biological advantages translate into durable clinical benefit and for whom.

## 5. Conclusions

In conclusion, the currently available comparative literature indicates that no statistically significant differences in outcomes were detected between ESS-based approaches for lumbar spondylolisthesis and heterogeneous non-endoscopic surgical strategies while offering possible perioperative advantages such as reduced blood loss and shorter hospitalization. However, these findings should be interpreted cautiously because the underlying evidence is predominantly retrospective, includes substantial heterogeneity in both ESS and comparator procedures, and spans markedly different follow-up durations. Therefore, the present analysis should be interpreted as indicating no statistically significant differences rather than equivalence and should be viewed as directionally informative rather than definitive evidence of comparative effectiveness, and higher-quality, more methodologically homogeneous studies are needed.

## Figures and Tables

**Table 1:** Summary of included studies.

Study ID	Study Design	Country	Type of Endoscopic Spine Procedures	Inclusion Criteria	Duration of Follow Up (Month)	Conclusion
Bahir 2024 [13]	Retrospective cohort	China	Unilateral Biportal Endoscopic Transforaminal Lumbar Interbody Fusion	Patients with single-segment degenerative lumbar spondylolisthesis accompanied by lumbar spinal stenosis (Meyerding grade I or II) with failed 3 months of conservative treatment.	24	UBE-TLIF and MIS-TLIF show similar effectiveness and fusion rates; UBE-TLIF reduces blood loss and hospital stay, while MIS-TLIF shortens operative time and fluoroscopy exposure.
Jin 2020 [14]	Retrospective cohort	China	Endoscopic-assisted lumbar interbody fusion	Patients with symptomatic low-grade lumbar spondylolisthesis (≤Grade II) and refractory to conservative treatment for ≥2 months	23.6 ± 6.6	PELIF minimizes iatrogenic damage with clinical and radiographic outcomes comparable to the minimally invasive surgery.
Guo 2023 [15]	Retrospective cohort	China	Unilateral Biportal Endoscopic Transforaminal Lumbar Interbody Fusion	Patients with single-level degenerative lumbar spondylolisthesis with lumbar spinal stenosis (LSS), Meyerding grade I/II, with failure of ≥3 months of conservative treatment.	24	BE-TLIF and MMIS-TLIF demonstrate comparable safety and effectiveness at mid-term follow-up, with UBE-TLIF showing advantages in reduced blood loss and quicker recovery.
Kao 2025 [16]	Retrospective cohort	Taiwan	Biportal endoscopic transforaminal lumbar interbody fusion	Patients with single-level degenerative lumbar spondylolisthesis	43	OLIF is superior in restoring lumbar lordosis and providing earlier relief of back pain, whereas BE-TLIF demonstrates comparable mid-term pain relief and fusion outcomes.
He 2022 [17]	Retrospective cohort	China	Percutaneous Endoscopic Posterior Lumbar Interbody Fusion with Unilateral Laminotomy for Bilateral Decompression	Patients with single-level degenerative lumbar spondylolisthesis with lumbar spinal stenosis (LSS) with failure of ≥3 months of conservative treatment.	18.4 ± 1.3	ULBD performed during PE-PLIF is a safe and effective minimally invasive technique. Compared with open PLIF, it reduces surgical invasiveness and accelerates postoperative recovery, although it requires a longer operative time
Kim 2021 [18]	Retrospective cohort	South Korea	Biportal Endoscopic Transforaminal Lumbar Interbody Fusion	Patients with single-level spondylolisthesis with spinal stenosis causing persistent neurological symptoms and intermittent claudication despite more than 3 months of conservative treatment.	18.4	BE-TLIF provides less early postoperative back pain, faster ambulation, and shorter hospital stay than MI-TLIF, with comparable long-term outcomes, fusion, and complication rates
Hua 2021 [19]	Retrospective cohort	China	Lumbar Endoscopic Unilateral Laminotomy for Bilateral Decompression	Patients with single-level LSS with Grade I degenerative spondylolisthesis refractory to >3 months of conservative management.	24	LE-ULBD and MI-TLIF are safe and effective. LE-ULBD is more minimally invasive with less blood loss, faster ambulation, and shorter hospital stay
Kimura 2019 [20]	Retrospective cohort	Japan	Microendoscopic Muscle-preserving Interlaminar Decompression	Patients had single-level, mild degenerative lumbar spondylolisthesis (Meyerding grade I without instability), failed ≥3 months of conservative treatment.	81	PLIF and ME-MILD achieved comparable long-term improvements in SF-36 and JOA scores, with no significant difference in revision surgery rates.
Li 2023 [21]	Retrospective cohort	China	Percutaneous Transforaminal Endoscopic Decompression	Patients had L4–L5 lumbar spinal stenosis with degenerative spondylolisthesis with no clinical improvement after ≥3 months of conservative treatment.	12	PTED demonstrated advantages in shorter operative time, less blood loss, smaller incisions, reduced drainage, shorter hospital stay, and fewer complications, and may serve as an effective alternative to MIS-TLIF.
Luo 2025 [22]	Retrospective cohort	China	Unilateral biportal endoscopic posterior lumbar interbody fusion	Patients with Meyerding grade I–II lumbar spondylolisthesis at L4–L5 with a slip distance > 3 mm, presenting with low back pain and bilateral radicular leg pain, and refractory to at least 12 weeks of conservative management.	≥12	UBE-PLIF offers advantages in minimal invasiveness, less drainage, faster low back pain relief, better muscle preservation, with comparable complication and fusion rates.
Lu 2024 [23]	Retrospective cohort	China	Unilateral biportal endoscopic transforaminal lumbar interbody fusion	Patients with single-segment Meyerding grade I lumbar spondylolisthesis who failed conservative treatment for approximately 3 months, had a follow-up period of more than 1 year, and presented with unilateral low back or leg pain.	13.7 ± 2.4	UBE-TLIF reduces postoperative drainage but has longer operative time.
Lv 2022 [24]	RCT	China	Endoscopic Transforaminal Lumbar Interbody Fusion	Patients with single-level Meyerding grade I lumbar spondylolisthesis presenting with unilateral radicular leg pain and who failed conservative therapy for at least 3 months.	18	Endo-TLIF is a safe, efficient, and minimally invasive alternative to MIS-TLIF, with satisfactory clinical outcomes.
Sim 2023 [25]	Retrospective cohort	Singapore	Endoscopic Transforaminal Lumbar Interbody Fusion	Patients with single-level degenerative disc disease associated with Meyerding grade I or II spondylolisthesis, mild to moderate central canal stenosis, and failure of conservative treatment.	≥12	E-TLIF is a safe and efficacious option that achieves clinical and radiological results similar to MIS-TLIF, with additional benefits of shorter surgery duration, less blood loss, and shorter hospital stay.
Yoshimizu 2025 [26]	Retrospective cohort	Japan	Biportal endoscopy-assisted extraforaminal lumbar interbody fusion	Patients with single-level L4–L5 degenerative spondylolisthesis.	24	BE-ELIF demonstrated superior spondylolisthesis reduction and disc height improvement than OLIF
Yun 2020 [27]	Retrospective cohort	South Korea	Posterolateral endoscopic lumbar discectomy	Patients aged 20–60 years with low-grade degenerative spondylolisthesis.	73 ± 26.6	PLELD is a good option to reduce recurrence and mitigate disc degeneration compared to OLM.
Zhang 2021 [28]	Retrospective case–control	China	Percutaneous endoscopic transforaminal lumbar interbody fusion	Patients aged >18 years with degenerative or isthmic spondylolisthesis (Meyerding grade I or II) associated with single-level instability, who failed at least 6 months of conservative therapy.	12	Endo-TLIF showed faster recovery and better early outcomes than MIS-TLIF
Zhou 2023 [29]	Retrospective cohort	China	Percutaneous transforaminal endoscopic surgery	Patients presenting with low back pain accompanied by leg symptoms, single-level lumbar spondylolisthesis at L2–L4 (Meyerding grade I or II), and failure of conservative treatment for at least 3 months.	24	PTES combined with mini-incision OLIF and anterolateral screws rod fixation is a good minimally invasive option with comparable long-term efficacy and safety to MIS-TLIF.
Zhu 2025 [30]	Retrospective cohort	China	Unilateral Biportal Endoscopic Lumbar Interbody Fusion	Patients with single-level Meyerding grade I degenerative lumbar spondylolisthesis and persistent symptoms for at least 3 months despite conservative treatment.	14.3 ± 1.5	Compared to MI-TLIF, ULIF has advantages of less hemorrhage, less inflammation, and earlier fusion, but a longer operation time.

BE-TLIF: Biportal Endoscopic Transforaminal Lumbar Interbody Fusion, E-TLIF: Endoscopic Transforaminal Lumbar Interbody Fusion, LSS: Lumbar Spinal Stenosis, MIS-TLIF: Minimally Invasive Transforaminal Lumbar Interbody Fusion, OLIF: Oblique Lumbar Interbody Fusion, RCT: Randomized Controlled Trial, TLIF: Transforaminal Lumbar Interbody Fusion, UBE: Unilateral Biportal Endoscopic, UBE-TLIF: Unilateral Biportal Endoscopic Transforaminal Lumbar Interbody Fusion.

**Table 2:** Baseline characteristics of included studies’ participants.

Study ID	Study Groups	Total Participants	Age (y), Mean (SD)	Male, *n* (%)	Meyerding Grade, *n* (%)		Type of Spondylolisthesis, *n* (%)		VAS Back, Mean (SD)	VAS Lower Extremities, Mean (SD)	ODI, Mean (SD)	Disc Height, Mean (SD)	Lumbar Lordosis Angle, Mean (SD)
					I	II	Isthmic Spondylolisthesis	Degenerative Spondylolisthesis					
Bahir 2024 [13]	Unilateral bi-portal endoscopic transforaminal lumbar interbody fusion	40	64.77 (5.68)	24 (60)	27 (67.5)	13 (32.5)	8 (20)	32 (80)	5.47 (0.90)	5.70 (1.47)	59.42 (6.45)	8.54 (0.46)	48.85 (5.96)
	Minimally invasive transforaminal lumbar interbody fusion	45	65.64 (5.21)	27 (60)	34 (75.5)	11 (24.4)	15 (33.3)	30 (66.6)	5.42 (0.75)	5.44 (1.43)	58.37 (6.01)	8.77 (0.55)	50.26 (6.66)
Jin 2020 [14]	Endoscopic-assisted lumbar interbody fusion	16	61.2 (8.9)	9 (56.3)	-	-	0	16 (100)	4.2 (1.5)	6.0 (1.2)	41.7 (4.8)	6.9 (1.1)	48.3 (8.4)
	Oblique lumbar interbody fusion	32	63.9 (7.6)	18 (56.3)	-	-	0	32 (100)	4.7 (1.7)	5.8 (1.4)	43.3 (3.0)	8.0 (2.0)	47.4 (9.6)
Guo 2023 [15]	Unilateral Biportal Endoscopic transforaminal lumbar interbody fusion	26	64.15 (6.42)	12 (46.2)	18 (69.2)	8 (30.8)	7 (26.9)	9 (73.1)	-	-	-	8.64 (0.59)	45.52 (1.69)
	3D Microscope-Assisted MIS-transforaminal lumbar interbody fusion	23	66.09 (6.10)	10 (43.5)	16 (69.6)	7 (30.4)	5 (21.7)	18 (78.3)	-	-	-	8.82 (0.55)	46.19 (1.39)
Kao 2025 [16]	Biportal endoscopic transforaminal lumbar interbody fusion	30	58.4 (10.2)	17 (56.7)	-	-	0	30 (100)	6.01 (1.52)	6.92 (1.21)	64.2 (12.81)	6.54 (1.56)	-
	Oblique lumbar interbody fusion	34	60.3 (11.9)	22 (64.7)	-	-	0	34 (100)	6.53 (1.14)	6.37 (1.11)	60.09 (5.99)	7.51 (1.33)	-
He 2022 [17]	Percutaneous Endoscopic Posterior Lumbar Interbody Fusion	28	59.8 (10.9)	14 (50)	11 (39.3)	17 (60.7)	-	-	4.61 (1.42)	6.29 (0.85)	47.36 (5.31)	8.66 (1.45)	35.36 (10.27)
	Open Posterior Lumbar Interbody Fusion	28	54.2 (10.3)	13 (46.4)	13 (46.4)	15 (53.6)	-	-	4.64 (1.39)	6.21 (0.88)	45.61 (3.87)	8.75 (1.65)	40.93 (7.09)
Kim 2021 [18]	Endoscopic Transforaminal Lumbar Interbody Fusion	32	70.5 (8.26)	17 (53.1)	-	-	6 (18.7)	26 (81.2)	7.9 (0.6)	7.9 (0.6)	68.1 (5.4)	-	-
	Minimally invasive transforaminal lumbar interbody fusion	55	67.3 (10.7)	25 (45.4)	-	-	7 (10.9)	48 (87.2)	7.8 (1.7)	7.8 (1.7)	69.6 (6.2)	-	-
Hua 2021 [19]	Lumbar Endoscopic Unilateral Laminotomy Bilateral Decompression	24	59.0 (7.9)	8 (33.3)	24 (100)	0	0	24 (100)	5.6 (0.9)	7.1 (0.7)	50.6 (3.2)	-	-
	Minimally Invasive Transforaminal Lumbar Interbody Fusion	36	59.9 (8.6)	10 (27.8)	36 (100)	0	0	36 (100)	5.8 (1.3)	7.3 (0.8)	51.2 (3.4)	-	-
Kimura 2019 [20]	Microendoscopic Muscle-preserving Interlaminar Decompression	28	70 (12.75)	11 (39.2)	28 (100)	0	0	28 (100)	-	-	-	-	-
	Posterior Lumbar Interbody Fusion	50	68.5 (9.25)	16 (32)	50 (100)	0	0	50 (100)	-	-	-	-	-
Li 2023 [21]	Percutaneous transforaminal endoscopic decompression	44	70.3 (7.5)	11 (25.0)	29 (65.9)	15 (34.1)	0	44 (100)		7.32 (1.07)	65.32 (9.29)	-	-
	Minimally invasive transforaminal lumbar interbody fusion	46	68.6 (6.0)	13 (28.3)	29 (63.0)	17 (37.0)	0	46 (100)		7.41 (1.05)	66.65 (9.23)	-	-
Luo 2025 [22]	Unilateral biportal endoscopic posterior lumbar interbody fusion	28	63.54 (6.62)	13 (46.4)	-	-	0	28 (100)	6.93 (0.71)	7.04 (0.69)	59.57 (6.17)	7.04 (0.96)	41.71 (8.21)
	Open Posterior Lumbar Interbody Fusion	31	62.23 (8.01)	13 (41.9)	-	-	0	31 (100)	7.06 (0.72)	7.06 (0.72)	56.90 (6.66)	6.97 (0.97)	40.03 (5.98)
Lu 2024 [23]	Unilateral biportal endoscopic transforaminal lumbar interbody fusion	26	59.0 (14.4)	18 (69.2)	26 (100)	0	-	-	6.5 (0.8)	7.2 (0.8)	63.8 (5.3)	8.92 (1.78)	32.35 (5.38)
	Minimally invasive transforaminal lumbar interbody fusion	28	61.7 (13.3)	18 (64.3)	28 (100)	0	-	-	6.7 (0.5)	7.3 (0.9)	62.3 (4.6)	8.82 (1.70)	30.75 (6.25)
	Open transforaminal lumbar interbody fusion	25	60.5 (13.9)	17 (68.0)	25 (100)	0	-	-	6.9 (0.7)	7.6 (0.5)	64.3 (6.5)	8.08 (1.32)	34.28 (7.17)
Lv 2022 [24]	Endoscopic Transforaminal Lumbar Interbody Fusion	54	-	-	54 (100)	0	-	-	7.51 (0.92)	7.59 (0.95)	57.3 (7.9)	-	-
	Minimally invasive transforaminal lumbar interbody fusion	48	-	-	48 (100)	0	-	-	7.82 (0.71)	8.11 (0.85)	62 (8.1)	-	-
Sim 2023 [25]	Endoscopic Transforaminal Lumbar Interbody Fusion	12	67.4 (7.1)	5 (41.7)	-	-	0	12 (100)	5.5 (3.0)	5.8 (3.0)	39.8 (14.1)	6.8 (1.5)	-
	Minimally invasive transforaminal lumbar interbody fusion	34	66.3 (9.9)	17 (50.0)	-	-	0	34 (100)	-	-	-	-	-
Yoshimizu 2025 [26]	Unilateral biportal endoscopy assisted extraforaminal lumbar interbody fusion	27	66.1 (10.7)	10 (37.0)	23 (85)	4 (15)	0	27 (100)	4.48 (3.0)	6.81 (2.9)	-	7.5 (1.7)	39.9 (10.3)
	Oblique lumbar interbody fusion	22	68.7 (9.2)	13 (59.1)	20 (91)	2 (9)	0	22 (100)	4.22 (3.0)	7.64 (2.3)	-	7.4 (2.1)	37.9 (10.6)
Yun 2020 [27]	Endoscopic Lumbar Discectomy	34	54.18 (9.87)	22 (64.7)	34 (100)	0	0	34 (100)	6.28 (2.5)	8.06 (1.87)	-	7.35 (1.76)	34.09 (24.31)
	Open Lumbar Microdiscectomy	32	53.29 (7.55)	16 (50)	32 (100)	0	0	32 (100)	4.32 (2.28)	6.28 (2.52)	-	7.38 (1.93)	37.96 (15.95)
Zhang 2021 [28]	Endoscopic Transforaminal Lumbar Interbody Fusion	32	53.1 (12.8)	12 (37.5)	22 (68.8)	10 (31.2)	14 (43.8)	19 (59.4)	6.84 (1.65)	7.38 (2.61)	40.52 (8.70)	-	-
	Minimally invasive transforaminal lumbar interbody fusion	30	55.7 (14.2)	14 (46.7)	24 (80.0)	6 (20.0)	18 (60.0)	12 (40.0)	6.52 (1.27)	7.88 (2.54)	42.16 (8.41)	-	-
Zhu 2025 [30]	Unilateral biportal endoscopy assisted extraforaminal lumbar interbody fusion	28	64.2 (5.4)	12 (42.9)	28 (100)	0	0	28 (100)	6.73 (0.95)	6.97 (1.21)	64.54 (3.40)	7.12 (1.37)	31.15 (9.55)
	Minimally invasive transforaminal lumbar interbody fusion	27	65.7 (4.8)	13 (48.1)	27 (100)	0	0	27 (100)	6.77 (1.00)	7.01 (1.24)	64.85 (2.92)	7.84 (1.83)	31.13 (8.78)
Zhou 2023 [29]	Percutaneous transforaminal endoscopic surgery	31	60 (8)	19 (61.3)	-	-	-	14 (45.2)	6.0 (1.5)	9.0 (0.75)	66.9 (8.8)	9.5 (1.7)	40.1 (9.7)
	minimally invasive surgery-transforaminal lumbar interbody fusion	34	61 (7)	13 (38.2)	-	-	-	12 (35.3)	6.0 (1.5)	8.0 (0.75)	67.2 (9.0)	9.6 (2.0)	40.0 (9.1)

ODI: Oswestry Disability Index, SD: Standard Deviation, VAS: Visual Analog Scale.

## Data Availability

The data supporting the findings of this systematic review and meta-analysis are derived from previously published studies cited in the manuscript. Extracted data used for the quantitative analyses are available within the article and its Supplementary Materials. No new individual participant data were generated during this study.

## Supplementary Materials

The following supporting information can be downloaded at https://www.mdpi.com/article/10.3390/jcm15124751/s1: Figure S1: Cochrane Risk of Bias 2 tool; Figure S2: Leave one out VAS back pain; Figure S3: Funnel plot of VAS back pain; Figure S4: Forest plot of VAS back pain based on comparator; Figure S5: leave one out VAS leg pain; Figure S6: Funnel plot of VAS leg pain; Figure S7: VAS leg pain based on comparator; Figure S8: Leave-one-out for Oswestry disability index; Figure S9: Funnel plot of ODI; Figure S10: forest plot of ODI based on comparator; Figure S11: Forest plot of blood loss; Figure S12: Forest plot of Hospital Stay (days); Figure S13: Forest plot of operative Time (minutes); Figure S14: Forest plot of postoperative complication; Figure S15. subgroup analysis of VAS back pain: Endoscopic Fusion vs. MIS Fusion; Figure S16. subgroup analysis of VAS leg pain: Endoscopic Fusion vs. MIS Fusion; Figure S17. subgroup analysis of ODI: Endoscopic Fusion vs. MIS Fusion; Table S1: PRISMA checklist; Table S2: Detailed search terms of each database; Table S3: Confounder assessment for each included study; Table S4: Risk of bias in observational studies using the Newcastle Ottawa Scale (NOS); Table S5: GRADE assessment; Table S6: Sensitivity analysis by Follow-up; Table S7: Summary of Fusion Definitions by Study; Table S8: Subgroup analysis: Endoscopic Fusion vs MIS Fusion (Primary Comparison); Table S9: Endoscopic Decompression vs Non-Endoscopic Decompression.

## Abbreviations

The following abbreviations are used in this manuscript:

CK	Creatine kinase
DH	Disc height
ERAS	Enhanced Recovery After Surgery
ESS	Endoscopic spine surgery
LLA	Lumbar lordosis angle
LLIF	Lateral lumbar interbody fusion
LOS	Length of stay
LSS	Lumbar spinal stenosis
MIS	Minimally invasive surgery
ODI	Oswestry Disability Index
OLIF	Oblique lumbar interbody fusion
OSS	Open spine surgery
PLIF	Posterior lumbar interbody fusion
TLIF	Transforaminal lumbar interbody fusion
UBE	Unilateral biportal endoscopic
VAS	Visual Analog Scale
