# Supplementary material for: Endoscopic Spine Surgery vs. Conventional Approaches for Lumbar Spondylolisthesis: Systematic Review and Meta-Analysis
Source: J Clin Med. 2026 Jun 18;15(12):4751. doi: 10.3390/jcm15124751 (PMC13300785; doi:10.3390/jcm15124751)
Supplement: Supplementary file 1 [file jcm-15-04751-s001.zip › jcm-4337742-supplementary.pdf]

Supplementary Table s1. PRISMA checklist

Section and Topic	Item #	Checklist item	Location where item is reported
**TITLE**			
Title	1	Identify the report as a systematic review.	1
**ABSTRACT**			
Abstract	2	See the PRISMA 2020 for Abstracts checklist.	1
**INTRODUCTION**			
Rationale	3	Describe the rationale for the review in the context of existing knowledge.	2
Objectives	4	Provide an explicit statement of the objective(s) or question(s) the review addresses.	2
**METHODS**			
Eligibility criteria	5	Specify the inclusion and exclusion criteria for the review and how studies were grouped for the syntheses.	3
Information sources	6	Specify all databases, registers, websites, organisations, reference lists and other sources searched or consulted to identify studies. Specify the date when each source was last searched or consulted.	
Search strategy	7	Present the full search strategies for all databases, registers and websites, including any filters and limits used.	3
Selection process	8	Specify the methods used to decide whether a study met the inclusion criteria of the review, including how many reviewers screened each record and each report retrieved, whether they worked independently, and if applicable, details of automation tools used in the process.	3,4
Data collection process	9	Specify the methods used to collect data from reports, including how many reviewers collected data from each report, whether they worked independently, any processes for obtaining or confirming data from study investigators, and if applicable, details of automation tools used in the process.	3,4
Data items	10a	List and define all outcomes for which data were sought. Specify whether all results that were compatible with each outcome domain in each study were sought (e.g. for all measures, time points, analyses), and if not, the methods used to decide which results to collect.	4
	10b	List and define all other variables for which data were sought (e.g. participant and intervention characteristics, funding sources). Describe any assumptions made about any missing or unclear information.	4
Study risk of bias assessment	11	Specify the methods used to assess risk of bias in the included studies, including details of the tool(s) used, how many reviewers assessed each study and whether they worked independently, and if applicable, details of automation tools used in the process.	4
Effect measures	12	Specify for each outcome the effect measure(s) (e.g. risk ratio, mean difference) used in the synthesis or presentation of results.	4
Synthesis methods	13a	Describe the processes used to decide which studies were eligible for each synthesis (e.g. tabulating the study intervention characteristics and comparing against the planned groups for each synthesis (item #5)).	3
	13b	Describe any methods required to prepare the data for presentation or synthesis, such as handling of missing summary statistics, or data conversions.	3,4
	13c	Describe any methods used to tabulate or visually display results of individual studies and syntheses.	3,4
	13d	Describe any methods used to synthesize results and provide a rationale for the choice(s). If meta-analysis was performed, describe the model(s), method(s) to identify the presence and extent of statistical heterogeneity, and software package(s) used.	4

Section and Topic	Item #	Checklist item	Location where item is reported
	13e	Describe any methods used to explore possible causes of heterogeneity among study results (e.g. subgroup analysis, meta-regression).	4
	13f	Describe any sensitivity analyses conducted to assess robustness of the synthesized results.	4
Reporting bias assessment	14	Describe any methods used to assess risk of bias due to missing results in a synthesis (arising from reporting biases).	4
Certainty assessment	15	Describe any methods used to assess certainty (or confidence) in the body of evidence for an outcome.	4
**RESULTS**			
Study selection	16a	Describe the results of the search and selection process, from the number of records identified in the search to the number of studies included in the review, ideally using a flow diagram.	4,5
	16b	Cite studies that might appear to meet the inclusion criteria, but which were excluded, and explain why they were excluded.	4,5
Study characteristics	17	Cite each included study and present its characteristics.	5-22
Risk of bias in studies	18	Present assessments of risk of bias for each included study.	22
Results of individual studies	19	For all outcomes, present, for each study: (a) summary statistics for each group (where appropriate) and (b) an effect estimate and its precision (e.g. confidence/credible interval), ideally using structured tables or plots.	5-22
Results of syntheses	20a	For each synthesis, briefly summarise the characteristics and risk of bias among contributing studies.	22-24
	20b	Present results of all statistical syntheses conducted. If meta-analysis was done, present for each the summary estimate and its precision (e.g. confidence/credible interval) and measures of statistical heterogeneity. If comparing groups, describe the direction of the effect.	22-25
	20c	Present results of all investigations of possible causes of heterogeneity among study results.	22-25
	20d	Present results of all sensitivity analyses conducted to assess the robustness of the synthesized results.	22-25
Reporting biases	21	Present assessments of risk of bias due to missing results (arising from reporting biases) for each synthesis assessed.	22-24
Certainty of evidence	22	Present assessments of certainty (or confidence) in the body of evidence for each outcome assessed.	22
**DISCUSSION**			
Discussion	23a	Provide a general interpretation of the results in the context of other evidence.	25,26
	23b	Discuss any limitations of the evidence included in the review.	26,27
	23c	Discuss any limitations of the review processes used.	26,27
	23d	Discuss implications of the results for practice, policy, and future research.	26
**OTHER INFORMATION**			
Registration and protocol	24a	Provide registration information for the review, including register name and registration number, or state that the review was not registered.	3
	24b	Indicate where the review protocol can be accessed, or state that a protocol was not prepared.	3
	24c	Describe and explain any amendments to information provided at registration or in the protocol.	3
Support	25	Describe sources of financial or non-financial support for the review, and the role of the funders or sponsors in the review.	28
Competing interests	26	Declare any competing interests of review authors.	28
Availability of data, code and other materials	27	Report which of the following are publicly available and where they can be found: template data collection forms; data extracted from included studies; data used for all analyses; analytic code; any other materials used in the review.	28

From: Page MJ, McKenzie JE, Bossuyt PM, Boutron I, Hoffmann TC, Mulrow CD, et al. The PRISMA 2020 statement: an updated guideline for reporting systematic reviews. BMJ 2021;372:n71. doi: 10.1136/bmj.n71. This work is licensed under CC BY 4.0. To view a copy of this license, visit https://creativecommons.org/licenses/by/4.0/

Supplementary Table S2: Detailed search terms of each database.

**Database**	Search	Number
**PubMed**	(endoscopy OR endoscopic) AND (spine OR spinal OR vertebral OR "vertebral column" OR "spinal column") AND (spondylolisthesis OR listhesis OR "vertebral slippage" OR "vertebral displacement" OR anterolisthesis OR retrolisthesis)	321
**Scopus**	TITLE-ABS-KEY (endoscopy OR endoscopic) AND (spine OR spinal OR vertebral OR "vertebral column" OR "spinal column") AND (spondylolisthesis OR listhesis OR "vertebral slippage" OR "vertebral displacement" OR anterolisthesis OR retrolisthesis)	488

**Web of Science**	(endoscopy OR endoscopic) AND (spine OR spinal OR vertebral OR "vertebral column" OR "spinal column") AND (spondylolisthesis OR listhesis OR "vertebral slippage" OR "vertebral displacement" OR anterolisthesis OR retrolisthesis) (Topic)	275
**Cochrane**	(endoscopy OR endoscopic) AND (spine OR spinal OR vertebral OR "vertebral column" OR "spinal column") AND (spondylolisthesis OR listhesis OR "vertebral slippage" OR "vertebral displacement" OR anterolisthesis OR retrolisthesis) in Title Abstract Keyword	33

Table S3: Confounder assessment for each included study.

**Study**	**Design**	**Slip Grade Reported**	**Spondylolisthesis Type**	**Baseline Symptoms**	**Age/Sex Balanced**
Bahir 2024	Retrospective cohort	Yes	Degenerative 80%	VAS, ODI	Acceptable
Jin 2020	Retrospective cohort	No	Degenerative 100%	VAS, ODI	Acceptable
Guo 2023	Retrospective cohort	Yes	Degenerative 73%	Disc height only	Acceptable
Kao 2025	Retrospective cohort	No	Degenerative 100%	VAS, ODI	Acceptable
He 2022	Retrospective cohort	Yes	Mixed	VAS, ODI	Acceptable
Kim 2021	Retrospective cohort	No	Degenerative 81%	VAS, ODI	Acceptable
Hua 2021	Retrospective cohort	Yes	Degenerative 100%	VAS, ODI	Acceptable

Kimura 2019	Case-control	Yes	Degenerative 100%	No baseline symptoms	Acceptable
Li 2023	Retrospective cohort	Yes	Degenerative 100%	VAS leg, ODI	Acceptable
Luo 2025	Retrospective cohort	No	Degenerative 100%	VAS, ODI	Acceptable
Lu 2024	Retrospective cohort	Yes	Degenerative 100%	VAS, ODI	Acceptable
Lv 2022	RCT	Yes	Degenerative 100%	VAS, ODI	Unclear
Sim 2023	Retrospective cohort	No	Degenerative 100%	VAS, ODI	Acceptable
Yoshimizu 2025	Retrospective cohort	Yes	Degenerative 100%	VAS	Acceptable
Yun 2020	Retrospective cohort	Yes	Degenerative 100%	VAS, disc height	Acceptable
Zhang 2021	Retrospective cohort	Yes	Mixed 59%	VAS, ODI	Acceptable
Zhu 2025	Retrospective cohort	Yes	Degenerative 100%	VAS, ODI	Acceptable
Zhou 2023	Retrospective cohort	No	Mixed 45%	VAS, ODI	Acceptable

Table S4: Risk of bias in observational studies using the Newcastle Ottawa Scale (NOS).

NOS for Cohort Studies								
	Selection				Comparability	Outcome		Quality Score
Study ID	Representativeness of the exposed cohort	Selection of the non-exposed cohort	Ascertainment of exposure	Demonstration that outcome of interest was not present at start of study	Comparability of cohorts on the basis of the design or analysis	Assessment of outcome	Was follow-up long enough for outcomes to occur	Adequacy of follow up of cohorts	
Bahir 2024	*	*	*	*	*	*	*	*	Good quality
Jin 2020	*	*	*		*	*	*	*	Good quality
Guo 2023	*		*	*	**	*	*		Good quality
Kao 2025	*	*	*	*	*	*	*		Good quality
He 2022	*	*	*	*	*	*	*	*	Good quality
Kim 2021	*	*	*	*	*	*	*	*	Good quality
Hua 2021	*	*	*	*	*	*	*	*	Good quality
Kimura 2019	*		*	*	*	*	*		Fair quality

Li 2023	*	*	*	*	*	*	*		Good quality
Luo 2025	*	*	*	*	*	*	*	*	Good quality
Lu 2024	*	*	*	*	*	*	*	*	Good quality
Sim 2023	*					*	*		Fair quality
Yoshimizu 2025	*	*	*	*	**	*	*	*	Good quality
Yun 2020	*		*	*	*	*	*		Fair quality
Zhang 2021	*	*	*	*	*	*	*	*	Good quality
Zhu 2025	*	*		*	*	*	*	*	Good quality
Zhou 2023 b	*	*	*	*	*	*	*	*	Good quality

Table S5: GRADE assessment

Outcome	n Studies (Design)	Risk of Bias	Inconsistency	Indirectness	Imprecision	Publication Bias	Certainty	Effect Estimate
**VAS Back Pain (change)**	13 (1 RCT, 12 obs)	Serious (−1)	Serious (−1)	No	Serious (−1)	Not detected	⨁◯◯◯ VERY LOW	MD -0.07 (95% CI: -0.33 to 0.18)
**VAS Leg Pain (change)**	14 (1 RCT, 13 obs)	Serious (−1)	Very serious (−2)	No	Serious (−1)	Not detected	⨁◯◯◯ VERY LOW	MD 0.08 (95% CI: -0.24 to 0.39)
**ODI (change)**	12 (1 RCT, 11 obs)	Serious (−1)	Serious (−1)	No	No	Not detected	⨁◯◯◯ VERY LOW	MD 0.51 (95% CI: -0.69 to 1.72)
**Blood Loss**	11 (1 RCT, 10 obs)	Serious (−1)	Very serious (−2)	No	No	Not detected	⨁◯◯◯ VERY LOW	MD -132.98 mL (95% CI: -227.97 to -37.99)
**Operative Time**	12 (1 RCT, 11 obs)	Serious (−1)	Very serious (−2)	No	No	Not detected	⨁◯◯◯ VERY LOW	MD 3.96 min (95% CI: -22.59 to 30.50)

**Hospital Stay**	11 (1 RCT, 10 obs)	Serious (−1)	Very serious (−2)	No	No	Not detected	⨁○○○ VERY LOW	MD -2.86 days (95% CI: -4.51 to -1.21)

Obs: observational studies; RCT: randomized controlled trial; MD; mean difference; RR; risk ratio; CI; confidence interval; **Starting certainty:** RCTs start at HIGH (⨁⨁⨁⨁); observational studies start at LOW (⨁⨁○○)

Table S6: Sensitivity analysis by Follow-up

**Short-term Follow-up (≤24 months): 15 studies**					
**Outcome**	**Studies**	**Pooled MD**	**95% CI**	**p-value**	**I^2**
VAS Back Pain	10	0.04	[-0.14, 0.21]	0.66	27.0%
VAS Leg Pain	11	0.20	[-0.08, 0.48]	0.16	73.0%
ODI	10	0.55	[-0.72, 1.81]	0.40	63.6%
Operation Time	12	+4.0 min	[-17.8, 25.8]	0.72	98.5%
Hospital Stay	11	-2.9 days	[-4.5, -1.2]	**0.0005**	96.9%
Blood Loss	10	-126.5 mL	[-162.8, -90.2]	**<0.0001**	98.5%
**Long-term Follow-up (>24 months): 3 studies (Kao 2025, Kimura 2019, Yun 2020)**					
**Outcome**	**Studies**	**Pooled MD**	**95% CI**	**p-value**	**I^2**
VAS Back Pain	2	-0.21	[-1.86, 1.44]	0.80	91.5%
VAS Leg Pain	2	-0.10	[-1.02, 0.83]	0.83	75.0%
ODI	1	Insufficient data for meta-analysis			
Other outcomes	0-1	Insufficient data for meta-analysis			

Table S7: Summary of Fusion Definitions by Study

**Study**	**Assessment Method**	**Definition of Successful Fusion**
**Bahir 2024**	X-ray, MRI, and CT	The sum of **fused and potentially fused cases** divided by the total number of cases.

**Guo 2023**	Radiography and CT	Use of the **Suk classification**; fusion rate calculated as (fusion cases + possible fusion cases) / total cases.
**He 2022**	X-ray or CT	Use of the **Bridwell criteria** evaluated at the last follow-up.
**Kao 2025**	X-ray	Presence of **trabeculae bridging** between the vertebral endplate and cage without a gap.
**Kim 2021**	Lateral and flexion-extension X-rays	**Trabecular bony bridge formation** and **< 4 degrees of segmental motion**; also utilized the Bridwell posterior fusion grade.
**Lu 2024**	CT scan	Use of the **Bridwell grading system** (specifically Grades I and II).
**Yoshimizu 2025**	CT scan	Presence of **bone formation between vertebral bodies** with continuous cephalocaudal endplates, facet joint fusion, and anterior or lateral cross-linking.
**Zhang 2021**	Dynamic X-ray and CT	**Clinical fusion:** < 4° movement and no mechanical low-back pain. **Standard fusion:** Continuous bone trabecular bridging between bodies.
**Zhu 2025**	CT scan	**Bridwell Grades I and II** (Grade I: remodeling and trabeculae; Grade II: intact graft with incomplete remodeling).
**Lv 2022**	CT scan	Ranked according to the **Brantigan criteria**, with **Grades 4 and 5** considered successful fusion.

Table S8: Subgroup analysis: Endoscopic Fusion vs MIS Fusion (Primary Comparison)

.

**Outcome**	**Studies (n)**	**Pooled MD**	**95% CI**	**p-value**	**I²**	**Interpretation**
**VAS Back Pain**	7	0.153	[-0.042, 0.348]	0.124	35.4%	No significant difference; moderate heterogeneity
**VAS Leg Pain**	7	0.001	[-0.384, 0.385]	0.997	81.4%	No significant difference; high heterogeneity
**ODI**	7	0.771	[-1.243, 2.784]	0.453	75.1%	No significant difference; high heterogeneity
**Operation Time**	7	17.152	[0.686, 33.619]	0.041	96.6%	Significantly longer for endoscopic fusion
**Hospital Stay**	7	-1.401	[-2.668, -0.133]	0.030	91.1%	Significantly shorter for endoscopic fusion
**Blood Loss**	5	-50.936	[-76.792, -25.079]	0.0001	97.1%	Significantly less for endoscopic fusion

Table S9: Endoscopic Decompression vs Non-Endoscopic Decompression

**Study**	**Outcome**	**MD**	**95% CI**
Kimura 2019	VAS Back	-0.21	[-0.81, 0.39]
Yun 2020	VAS Back	-1.07	[-1.88, -0.26]
Kimura 2019	VAS Leg	-0.42	[-1.11, 0.27]
Yun 2020	VAS Leg	-0.43	[-1.27, 0.41]

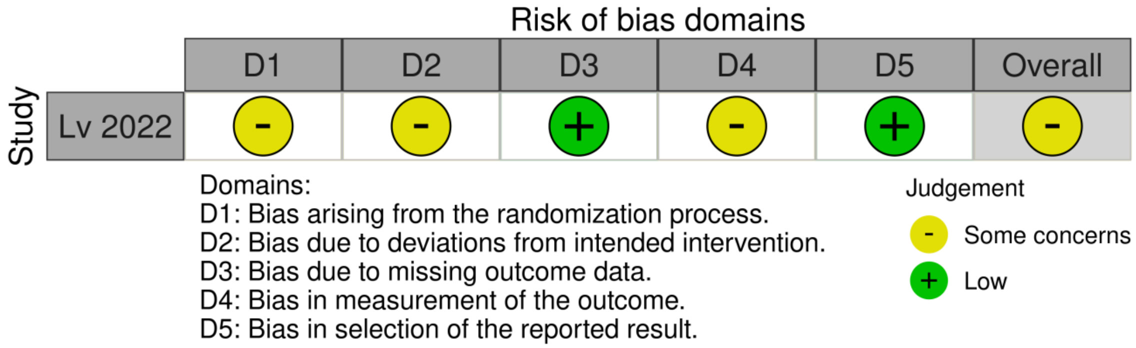

Supplementary Figure S1: Risk of Bias domains of the included randomized controlled trial based on the Cochrane Risk of Bias 2 tool

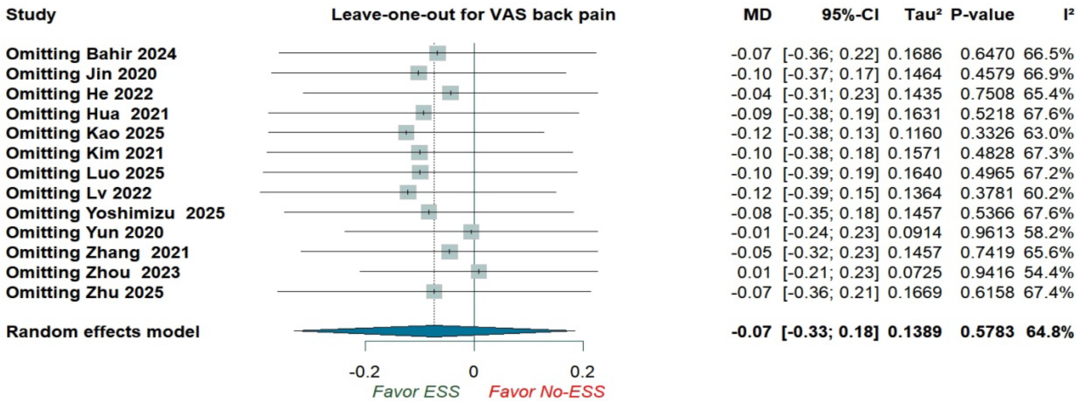

Supplementary Figure S2: Leave one out VAS back pain.

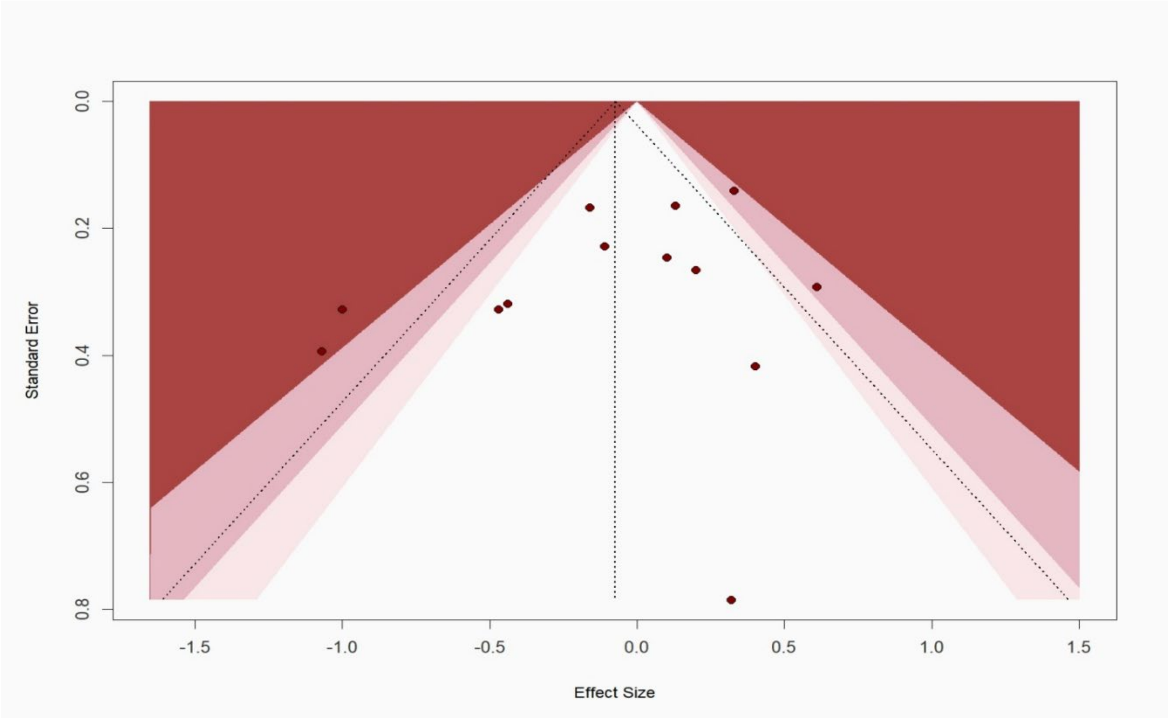

Supplementary Figure S3: Funnel plot of VAS back pain.

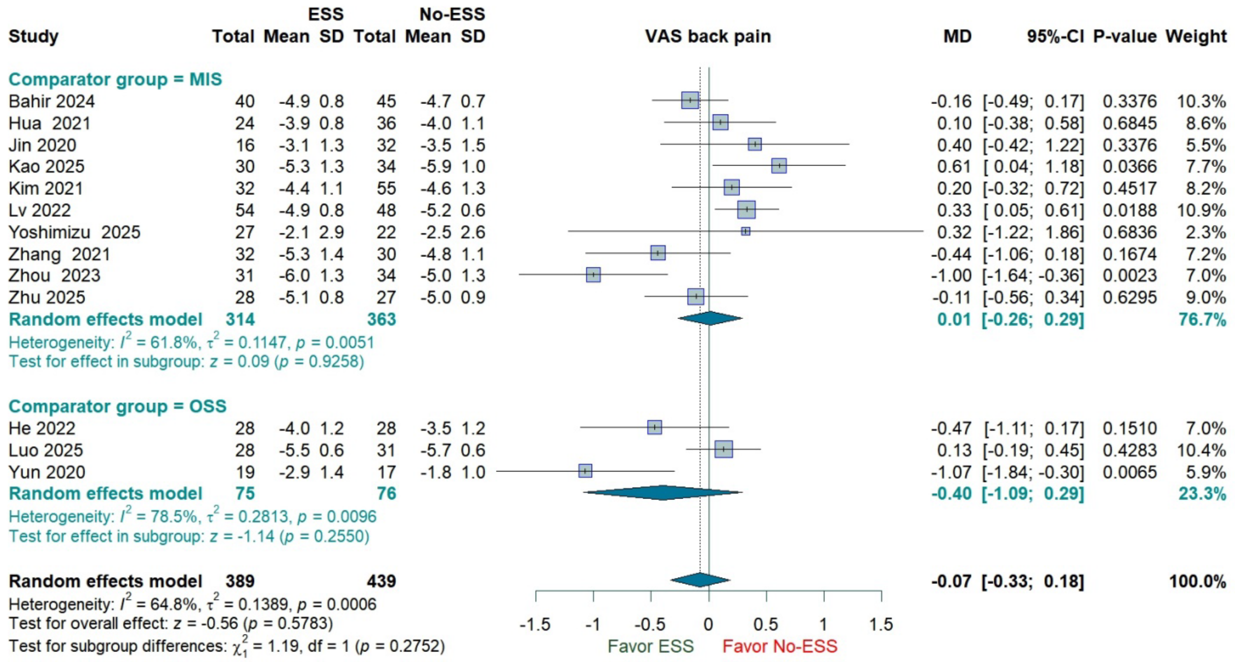

Supplementary Figure S4: Forest plot of VAS back pain based on comparator.

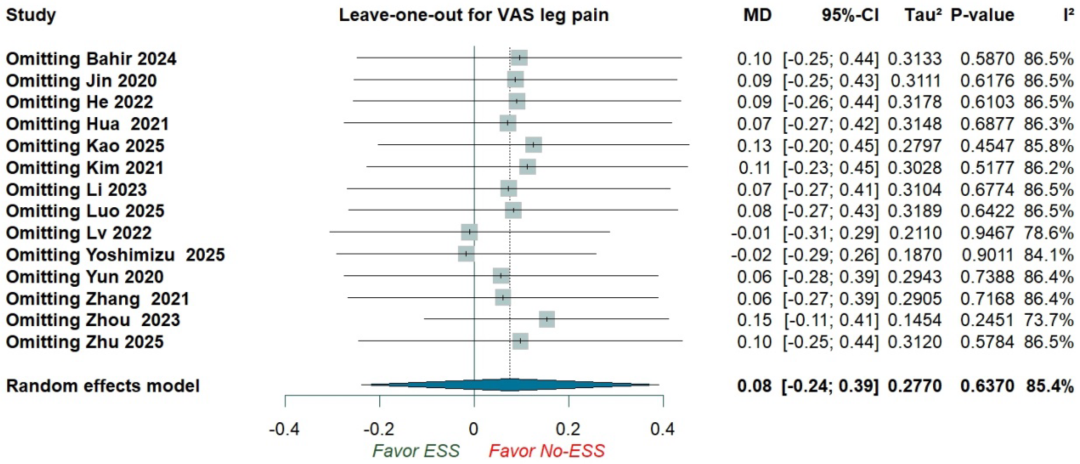

Supplementary Figure S5: leave one out VAS leg pain.

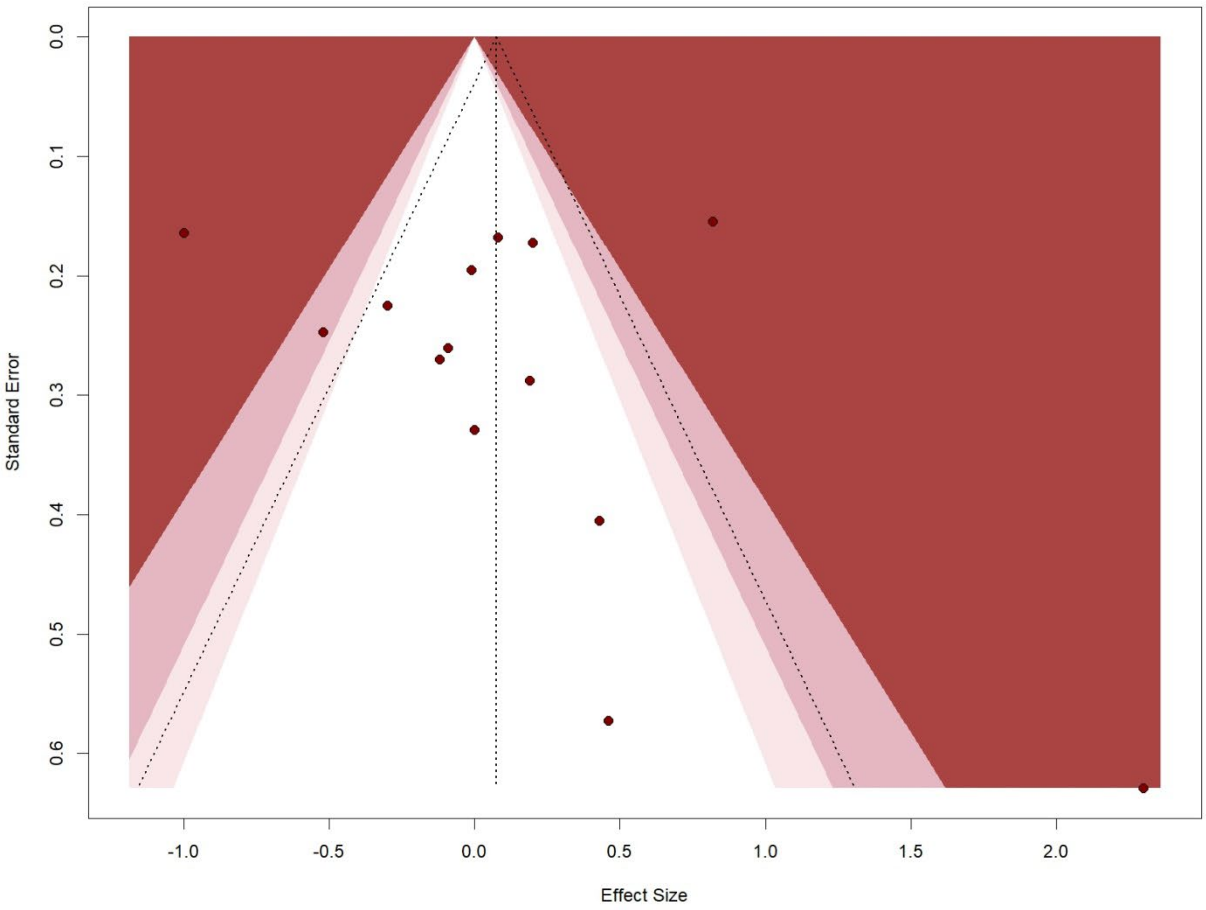

Supplementary Figure S6: Funnel plot of VAS leg pain.

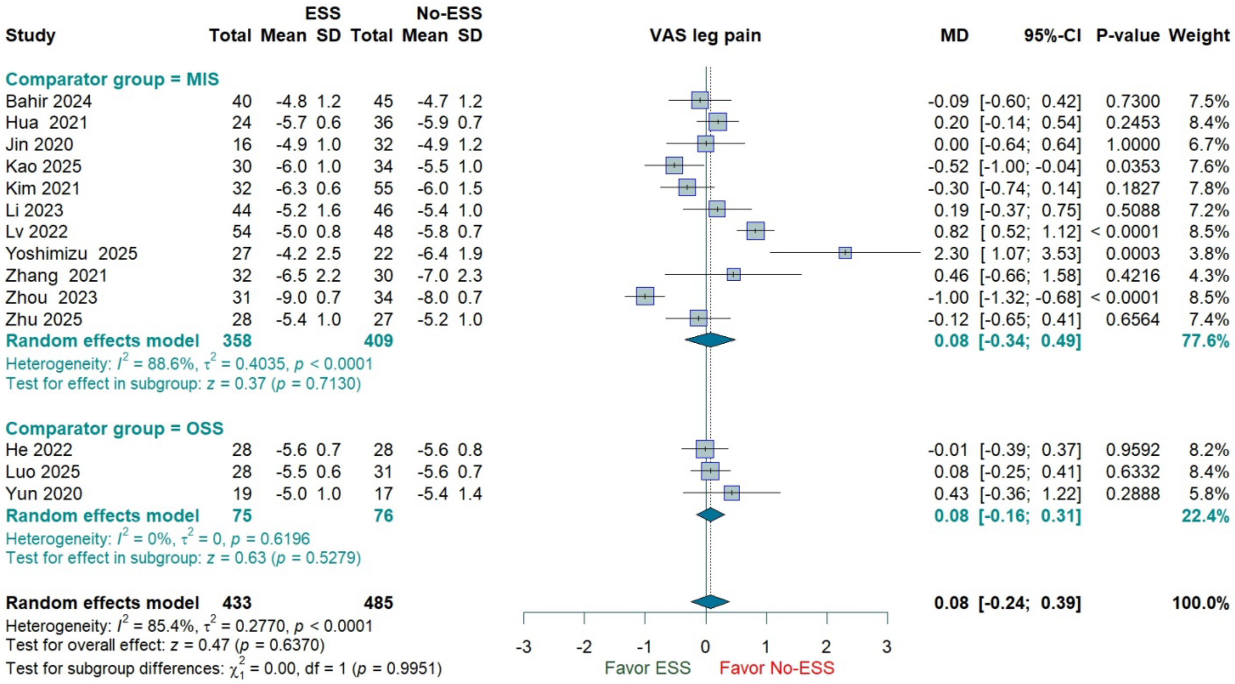

Supplementary Figure S7: VAS leg pain based on comparator.

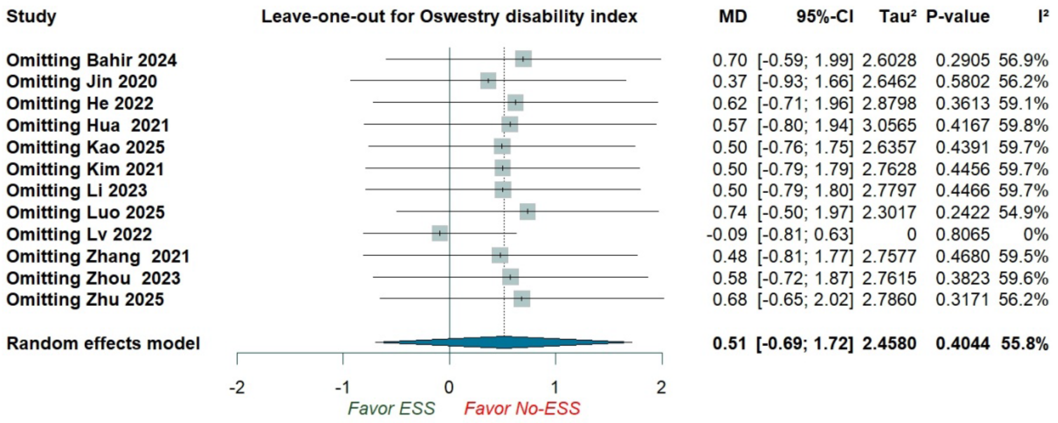

Supplementary Figure S8: Leave-one-out for Oswestry disability index.

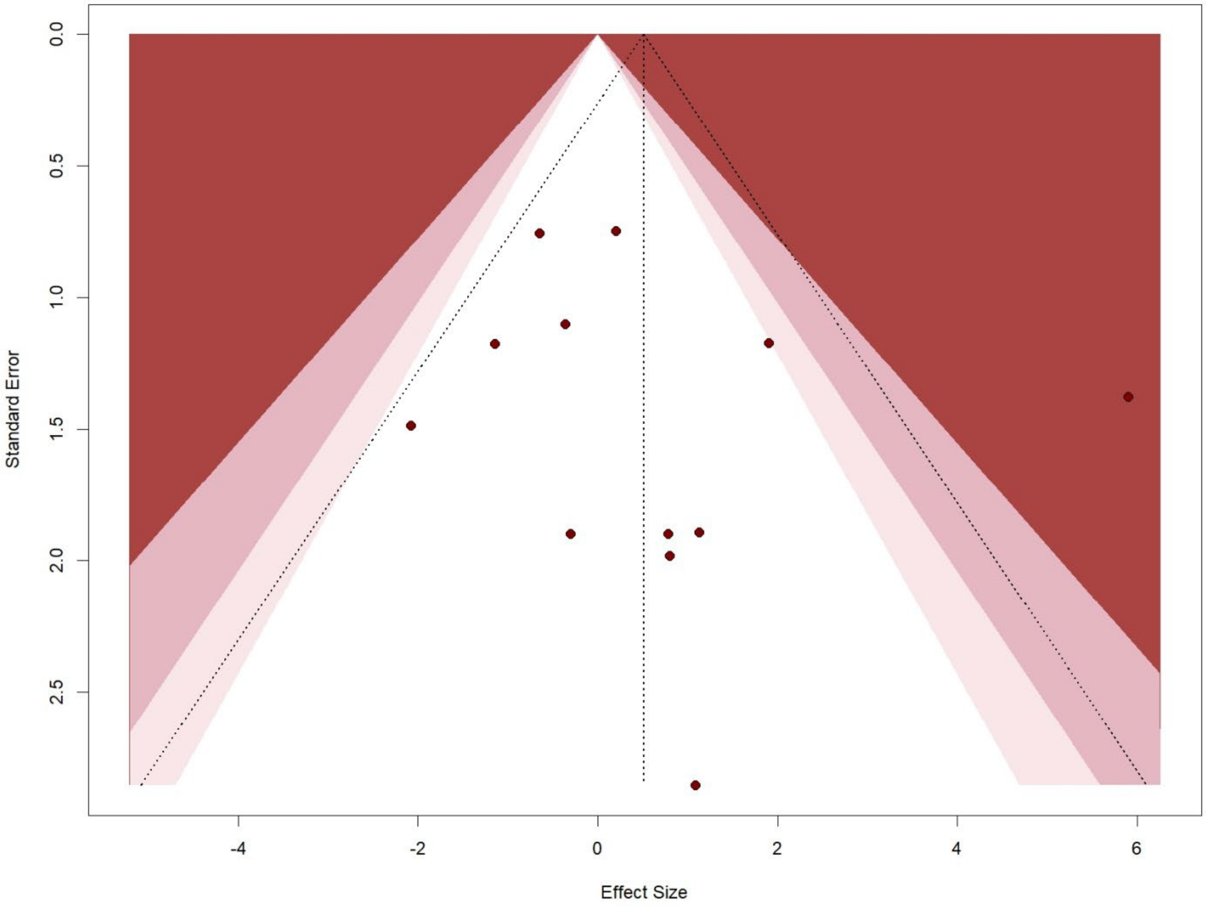

Supplementary Figure S9 Funnel plot of ODI.

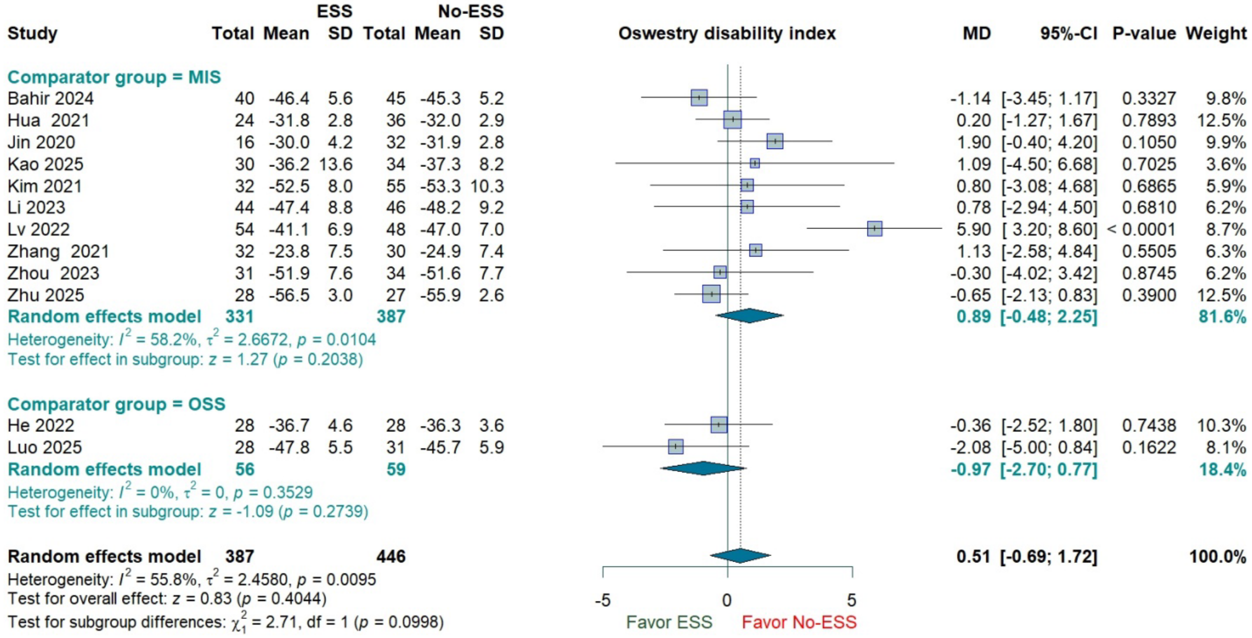

Supplementary Figure S10: forest plot of ODI based on comparator.

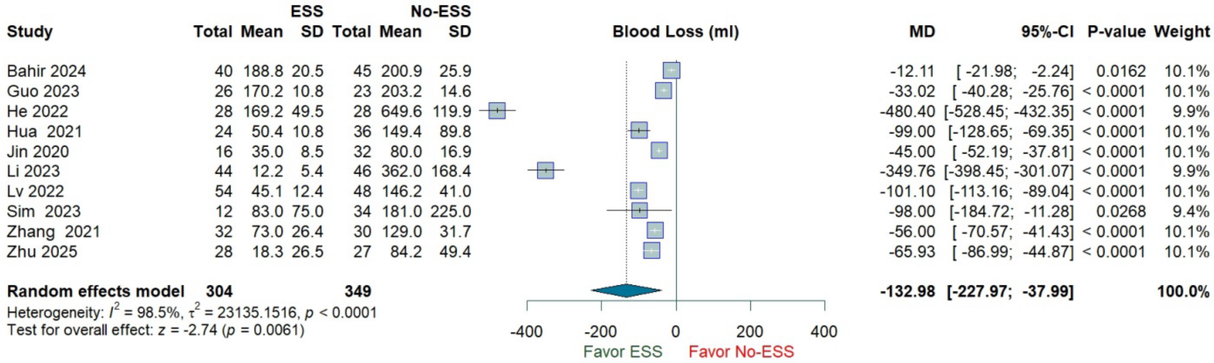

Supplementary Figure S11: Forest plot of blood loss.

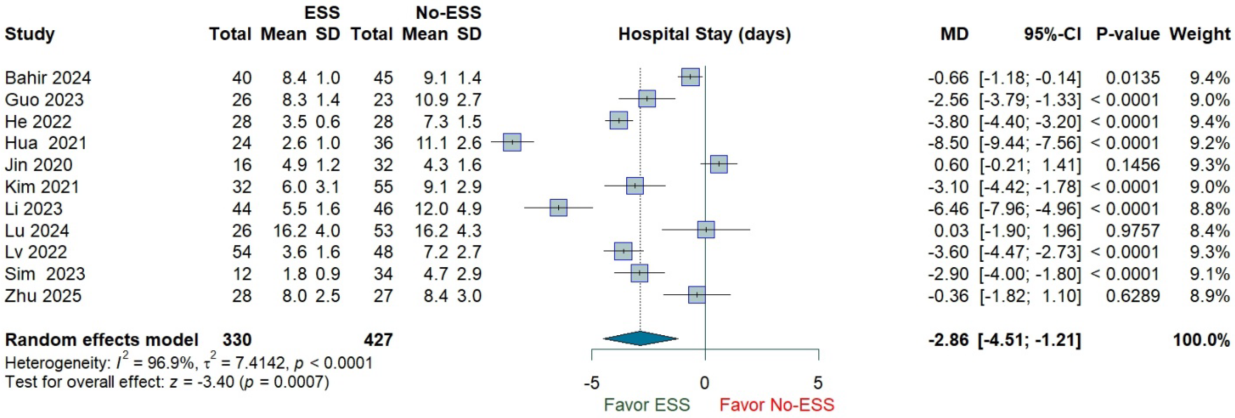

Supplementary Figure S12: Forest plot of Hospital Stay (days).

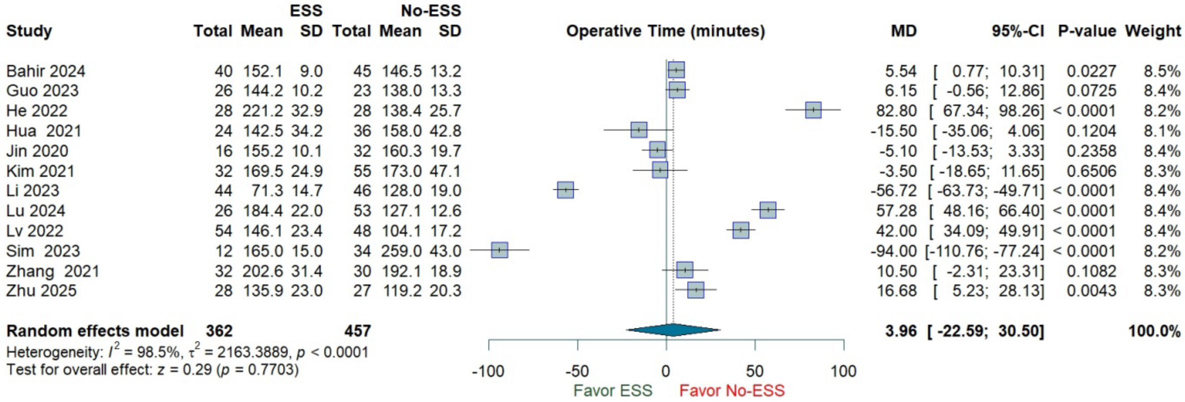

Supplementary Figure S13: Forest plot of operative Time (minutes).

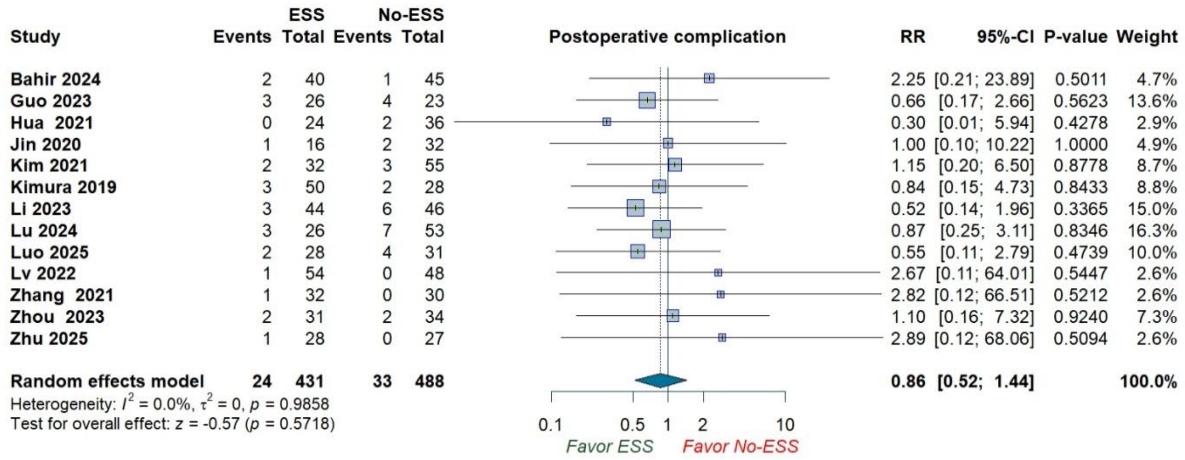

Supplementary Figure S14: Forest plot of postoperative complication

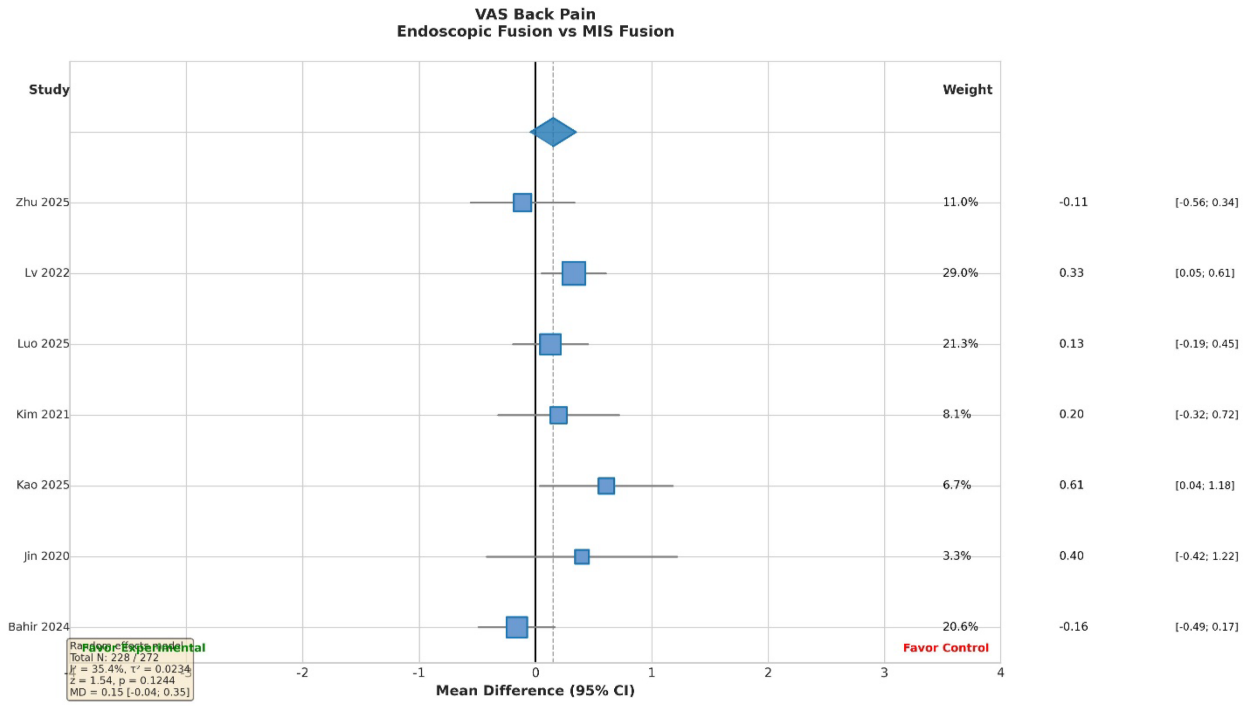

Supplementary Figure S15. subgroup analysis of VAS back pain: Endoscopic Fusion vs MIS Fusion.

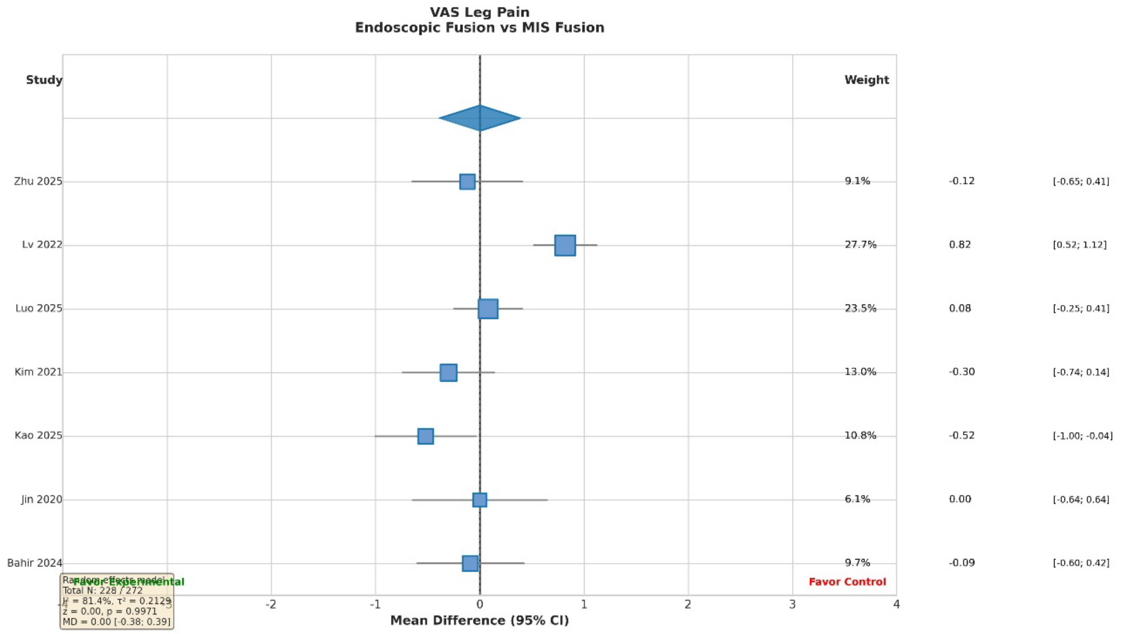

Supplementary Figure S16. subgroup analysis of VAS leg pain: Endoscopic Fusion vs MIS Fusion.

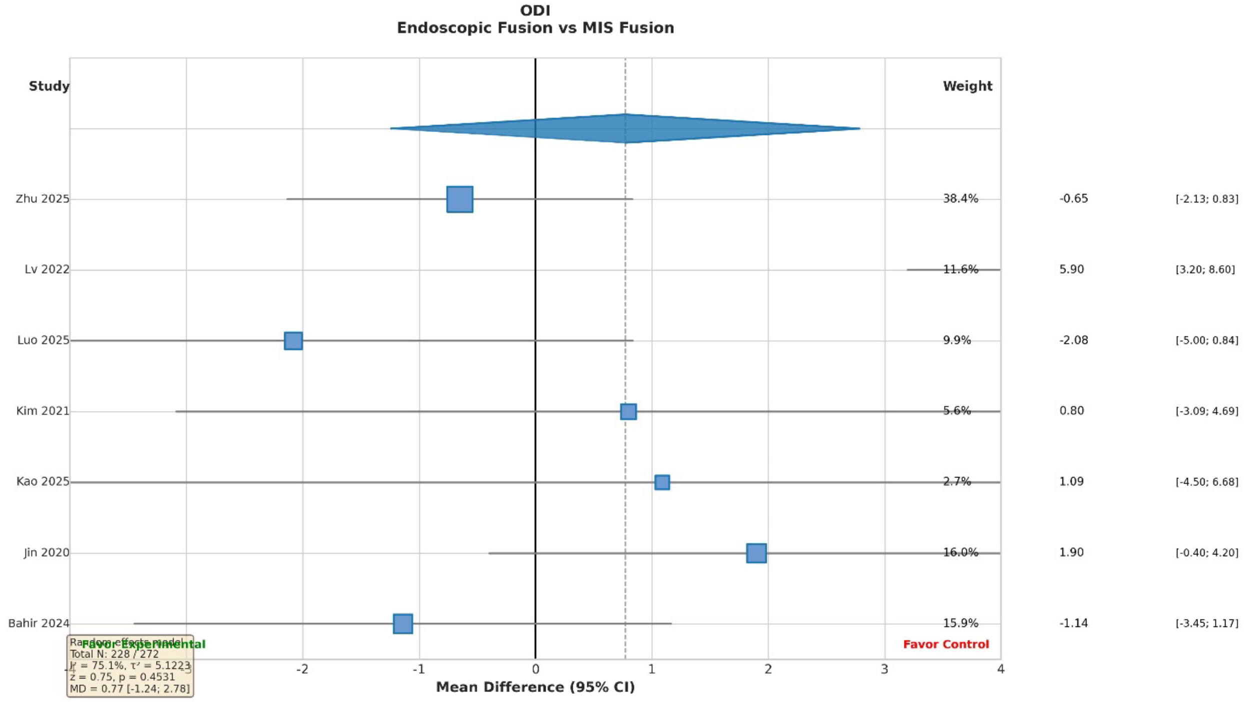

Supplementary Figure S17. subgroup analysis of ODI: Endoscopic Fusion vs MIS Fusion.
